# Ancestral Inference and the Study of Codon Bias Evolution: Implications for Molecular Evolutionary Analyses of the *Drosophila melanogaster* Subgroup

**DOI:** 10.1371/journal.pone.0001065

**Published:** 2007-10-24

**Authors:** Hiroshi Akashi, Piyush Goel, Anoop John

**Affiliations:** Institute of Molecular Evolutionary Genetics, Department of Biology, Pennsylvania State University, State College, Pennsylvania, United States of America; Washington University in St. Louis School of Medicine, United States of America

## Abstract

Reliable inference of ancestral sequences can be critical to identifying both patterns and causes of molecular evolution. Robustness of ancestral inference is often assumed among closely related species, but tests of this assumption have been limited. Here, we examine the performance of inference methods for data simulated under scenarios of codon bias evolution within the *Drosophila melanogaster* subgroup. Genome sequence data for multiple, closely related species within this subgroup make it an important system for studying molecular evolutionary genetics. The effects of asymmetric and lineage-specific substitution rates (*i.e*., varying levels of codon usage bias and departures from equilibrium) on the reliability of ancestral codon usage was investigated. Maximum parsimony inference, which has been widely employed in analyses of *Drosophila* codon bias evolution, was compared to an approach that attempts to account for uncertainty in ancestral inference by weighting ancestral reconstructions by their posterior probabilities. The latter approach employs maximum likelihood estimation of rate and base composition parameters. For equilibrium and most non-equilibrium scenarios that were investigated, the probabilistic method appears to generate reliable ancestral codon bias inferences for molecular evolutionary studies within the *D. melanogaster* subgroup. These reconstructions are more reliable than parsimony inference, especially when codon usage is strongly skewed. However, inference biases are considerable for both methods under particular departures from stationarity (*i.e*., when adaptive evolution is prevalent). Reliability of inference can be sensitive to branch lengths, asymmetry in substitution rates, and the locations and nature of lineage-specific processes within a gene tree. Inference reliability, even among closely related species, can be strongly affected by (potentially unknown) patterns of molecular evolution in lineages ancestral to those of interest.

## Introduction

Inference of ancestral and derived nucleotides within populations or among lineages is a critical step in a number of approaches to identify mechanisms of molecular evolution. Ancestral state inference has been employed to reveal episodic, or lineage-specific base composition and protein evolution [*e.g*.], [01], [02], [03], [04], [05], [06, 07]. In addition, several population genetic tests rely on ancestral reconstructions to reveal the action of natural selection on functional classes of mutations or in particular genetic regions [*e.g*.08], [09], [10], [11], [12], [13].

This study addresses the accuracy of ancestral codon usage inference using the phylogenetic relationships and distances among species in the *Drosophila melanogaster* subgroup as a model tree. This group of species has been the focus of a large number of studies of mechanisms of molecular evolution. In addition, genome sequences are now available for five species in this subgroup and large-scale polymorphism studies are underway for at least two species, *D. melanogaster* and *D. simulans*. Most studies that have incorporated ancestral inference in the *D. melanogaster* subgroup have employed maximum parsimony because it is simple to implement and is assumed to be accurate among closely related lineages [*e.g*.08], [14], [06], [15], [16], [17], [18]. However, parsimony inference can be biased under non-random character state usage (*i.e*., base composition bias) and long branch lengths [19]. Genome-wide declines of GC content have been inferred in a number of Drosophila lineages [02], [20], [21], [22], [23], [15], [24], [25], [26], [27], [16] as well as among mammals [28], [29], [30], [31], [32]; [33]. Support for increases in GC content in Drosophila are confined to specific genes or small genetic regions and/or a limited number of lineages [06], [34], [35], [36], [37]. Some studies have employed inference methods that account for rate variation among types of nucleotide changes or among lineages, but many claims of base composition differentiation are based solely on parsimony reconstructions. Because parsimony is biased toward inference of changes from common to rare states, genes whose base composition is skewed toward GC can show apparent declines of GC in the absence of actual changes in base composition [19], [38], [39], [40], [41].

Here, we employ computer simulations to determine the reliability of ancestral codon usage inference under parsimony and likelihood approaches that have been employed in studies of Drosophila codon bias evolution. The likelihood method implements the HKY85 model [42] which includes parameters for base composition bias. This study begins with a description of “major codon preference”, a model of weak selection at silent sites. Sequence data generated by computer simulations of major codon preference were used to determine the magnitude and direction of ancestral inference biases. Factors examined include levels of codon bias, branch lengths, departures from equilibrium, and the location of lineages within a phylogeny. We show that the direction and magnitude of biases in ancestral inference are dependent on all of these factors and are relevant to the study of molecular evolution among closely related species within the *D. melanogaster* subgroup. More general implications for inferring ancestral sequences under asymmetric and/or fluctuating substitution rates are discussed.

## Methods and Results

### Major codon preference

Synonymous codon usage appears to evolve under a balance among weak evolutionary forces in Drosophila and in a wide range of taxa [reviewed in 43], [44], [45], [46], [47], [48]. Codons that are used preferentially in highly expressed genes tend to be recognized by abundant tRNA isoacceptors [43], [49], [50], [51], [52], [53]. Such codons are termed “preferred” or “major” codons. Major codon usage is defined as the overall percentage of major codons at redundant codons in a gene, MCU = #major codons/(#major+#minor codons). MCU is positively correlated with gene expression levels [reviewed in 47] and biochemical studies have shown faster and more accurate translation at major codons than at other codons that encode the same amino acid, termed “minor” or “unpreferred” codons [reviewed in 45], [54]. Major codon preference posits that translationally superior codons confer a fitness advantage sufficient to bias codon usage (especially in highly expressed genes) but small enough to allow minor codons to persist through mutation pressure and genetic drift. Within-and between-species comparisons of silent mutations in a number of Drosophila species are consistent with small fitness benefits to major codon usage [08], [55], [14], [56], [15], [26], [57], [58], [17].

Under major codon preference, the evolutionary dynamics of synonymous changes are determined by the joint effects of mutation, genetic drift, and weak selection. Consider a two-fold redundant codon where A_1_ represents a major codon (relative fitness = 1) and A_2_ represents a minor codon (relative fitness = 1−*s*). Mutations may occur at different rates *u*, from A_1_ to A_2_, and *v*, in the reverse direction. A_1_ to A_2 _changes will be designated “*pu*” (for preferred to unpreferred) and A_2_ to A_1 _changes will be referred to as “*up*” (for unpreferred to preferred). At a locus consisting of a number of such codons, the frequency of major codons at the locus, MCU, will reach a steady state if *u*/*v* and *N*_e_ *s* remain constant over a large number of generations (see Methods S1 for details). However, small changes in parameter values can cause departures from steady-state that are both strong and long-lasting; new equilibria are approached on a time scale on the order of the reciprocal of *per* site mutation rate (10^8^–10^9^ generations in Drosophila). If changes in mutation rates or selection intensity occur on a faster time scale than the approach to equilibrium, codon usage may rarely be at steady-state.

**Figure 1:**
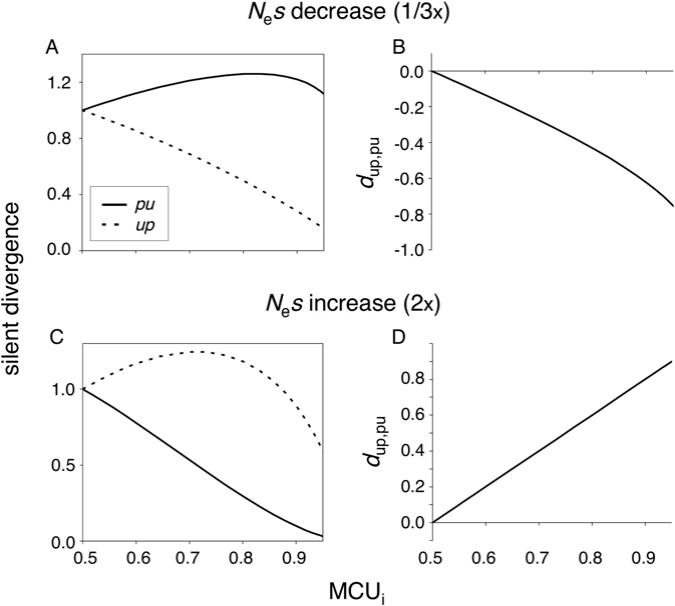
Silent divergence under departures from equilibrium major codon usage. The x-axis shows MCU values prior to a change in *N*_e_. A: expected instantaneous *per* locus rates of *pu* and *up* fixations when *N*_e_ decreases to 1/3 its original value across genes. B: expected *d*_up,pu_ = (#*up*−#*pu*)/(#*up*+#*pu*) for the 1/3*N*_e_ scenario (decreasing codon bias). C and D: expected instantaneous silent rates and *d*_up,pu_ after a doubling of *N*_e_ (increasing codon bias). Legends and X-axis scales apply to graphs in the same column. See text for details of the model. The curves assume that variation in selection coefficients underlies MCU variation among genes (*u/v* = 1 across genes).

Identifying lineage-specific codon bias changes and their causes is important to our understanding of evolution at synonymous sites and may help to reveal mechanisms of protein evolution [02]. Figures 1A and C show expected rates of *up* and *pu* substitutions following three-fold decreases and two-fold increases in *N*_e_, respectively. These *N*_e_ ratios give approximately two-fold excesses of *pu* and *up* changes, respectively, for a gene with initial MCU = 0.7. Changes in *N*_e_ are assumed to be constant across loci (*i.e*., in Figures 1A and B, all loci experience a three-fold decrease from their initial *N*_e_ *s* values). A measure of skew, *d*_up,pu_ = (#*up*–#*pu*)/(#*up*+#*pu*), is employed as an index of the direction and magnitude of departures from steady-state. Under non-stationary *N*_e_ *s*, expected *d*_up,pu_ varies considerably as a function of MCU.

Mutational variation can also cause genome-wide changes in codon bias. Because fixation rates are linearly dependent on mutation rates, the *pu*:*up* substitution ratio will reflect the altered ratio of *per* locus mutation rates. If codon bias variation among lineages is caused by uniform parameter changes, then comparisons of *d*_up,pu_ among codons experiencing different selection intensities (*i.e*., codons in different genes or regions within genes or in different synonymous families) can identify whether changes in scaled selection coefficients or mutational biases underlie codon bias differences. It should be noted, however, that the predictions above are based on a model that does not account for ancestral polymorphism. When *N*_e_ *u* is large, the frequency distribution of silent polymorphisms may need to be considered when predicting *up*:*pu* ratios following parameter changes. McVean and Charlesworth [59] showed that equilibrium MCU predictions are robust to polymorphism, but this result has not been confirmed for non-stationary evolution.

### Simulations of codon bias evolution

Computer simulations were employed to test the performance of ancestral reconstruction methods with a focus on inference of non-stationary codon bias and its causes in the *D. melanogaster* subgroup.

#### Tree topology and branch lengths

**Figure 2:**
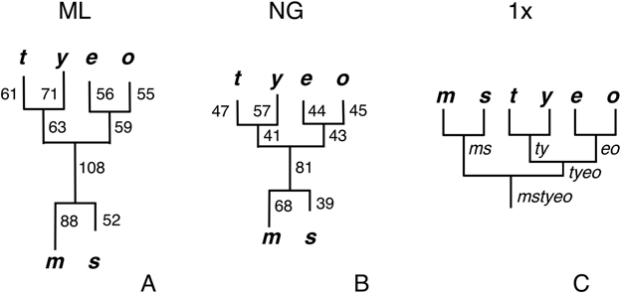
Synonymous distance trees for six *Drosophila melanogaster* subgroup species. *m, s, t, y, e,* and *o* refer to *D. melanogaster, D. simulans, D. teissieri, D. yakuba, D. erecta,* and *D. orena*, respectively. The assumed tree topology ((*m, s*), ((*t, y*), (*e, o*))) is based on [60]. Silent distances were calculated using CODEML [63] and averaged across 22 genes. (ML) unrooted tree showing maximum likelihood distances under a codon-based substitution model. Equilibrium frequencies of each codon were calculated from the nucleotides frequencies at three codon positions (F3x4). (NG) unrooted neighbor-joining tree based on Nei-Gojobori [64] pairwise distances. Numbers shown on each branch are per site synonymous distances x1000. (1x) topology employed in 1x simulations. Mutation rates and numbers of generations per branch were set to give expected *per* site silent divergence of 0.05 for the *m, s, t, y, e, o, ty*, and *eo* lineages and 0.075 and 0.025 for the *ms* and *tyeo* lineages, respectively, for equilibrium MCU = 0.7. Abbreviations for ancestral nodes are shown below and to the right of the nodes.

Data were simulated for six extant nodes given a topology and branch lengths set to estimates for the *D*. *melanogaster* subgroup (Figure 2). This tree topology is strongly supported in analyses of nuclear genes using sequences from relatively closely related outgroup species from the *takahashii* and *suzukii* subgroups [60], [61]. Analyses of much larger numbers of loci (but with more distantly related outgroups) are generally consistent with this tree, but also support gene-specific topologies that vary with respect to the placement of the *D. yakuba* and *D. erecta* clades [62].

Average synonymous divergence is shown for six species in the *D. melanogaster* subgroup in Figure 2. The branch lengths in the “1x” tree in Figure 2 were employed in the simulations. Extant nodes on the simulated tree will be referred to by the first letter of the species' name. Internal nodes are named according to their child nodes (*i.e*., the node connecting *t* and *y* is “*ty*” and the node connecting *ty* and *eo* is “*tyeo*”). Lineages will be referred to by the name of the upper node (*i.e*., “*m*” refers to the branch connecting the *ms* and *m* nodes and “*ms”* refers to the branch between the *mstyeo* and *ms* nodes). Branch lengths were chosen to give a symmetric (unrooted) tree with silent divergence levels between maximum likelihood [63] and Nei-Gojobori (NG) [64] estimates (see Figure 2 legend for details). Although data were simulated for the ancestral lineage (*mstyeo*) as well as the ten lineages in the 1x tree shown in Figure 2, ancestral states were reconstructed on an unrooted tree. Thus, silent changes were assigned to eight lineages: *m*, *s*, *t*, *y*, *ty*, *e*, *o*, and *eo*.

#### Simulating sequence evolution

Nucleotide changes were simulated for sequences with random amino acid usage (*i.e*., each amino acid was represented in the sequence in proportion to its level of redundancy in the standard genetic code). Sequence lengths were 4,941 (61×81) codons. Only synonymous changes at third codon positions were simulated (six-fold redundant codons evolved as two-fold or four-fold families). For each codon, state-transition or “substitution” probabilities to other synonymous codons were calculated by multiplying a *per* site *per* generation mutation rate of 2×10^−5^ and the fixation probability for the mutation. Codon changes in the simulated trees will be referred to as “fixations” or “substitutions” although population data were not simulated. Equal mutation rates were assumed among all nucleotides. All G- and C-ending synonymous codons were assigned a fitness of 1 and all A- and T-ending codons were assigned a fitness of 1-*s*. *N*_e_ was set to 5,000 diploid individuals and values of *s* were assigned to give the following equilibrium MCU values: 0.5, 0.6, 0.7, 0.8, 0.9, and 0.95 (in these simulations, MCU is equivalent to %GC). Nucleotide ambiguity codes S and W will be used to refer to G or C and A or T, respectively. This substitution model will be referred to as the “GCpref codon” model.

Variable parameters in the simulations were: the numbers of generations on each branch, mutation rates, the fitness advantage of G- or C-ending codons, *s*, and effective population size, *N*_e_. The tree topology was not varied among simulations. All simulations were initiated with a “burn-in” period of ≥2/*u* generations to insure independence among replicates (longer burn-in periods were used for high MCU scenarios). A total tree length of 9,000 generations from the *mstyeo* node gave distances of approximately 5% synonymous divergence on the *m*, *s*, *t*, *y*, *e*, *o*, *ty*, and *eo* lineages for the equilibrium MCU = 0.7 case. Simulations under this set of parameter values will be referred to as the “1x” scenario. Data were also produced for trees with the same topology but double (2×) and half (0.5×) the numbers of generations on each branch.

Non-equilibrium codon usage was simulated by adjusting effective population size or mutation parameters at the *mstyeo* node following the burn-in. Both increasing codon bias (larger *N*_e_ or reduced *u*/*v*) and decreasing bias (smaller *N*_e_ or elevated *u*/*v*) were simulated. Values of 2× and 1/3× the initial *N*_e_ and 1/2× and 2× the initial *u*/*v* were chosen to give *pu*:*up* fixation ratios of approximately half and double the equilibrium values for an initial MCU of 0.7. All parameters were held constant following the change at the ancestral node. For all simulations, the lineage and fitness class of each fixation was recorded and the resulting extant sequences were stored. Extant sequences were employed to generate inferences of the numbers and types of changes for comparisons to the numbers of “actual” (simulated) changes.

### Reliability of Ancestral Inference

Simulated sequence data were analyzed using BASEML in the PAML software package (Version 2.0k) [63]. This program takes a gene tree and sequence data as input and determines maximum likelihood estimates for branch lengths and parameters of a substitution model. These MLEs are employed in calculations of the posterior probabilities of joint reconstructions of nucleotides at ancestral nodes [65]. Two substitution models were employed for inferences of ancestral states among simulated sequences. Maximum parsimony (MP) inference was emulated using a Jukes-Cantor [66] one parameter model with all branch lengths set to small and equal values. Under this model, all nucleotide changes occur at equal rates and the set of ancestral nucleotides (joint reconstruction) that requires the fewest number of changes in the tree has the highest probability. A unique joint reconstruction requiring the lowest number of changes was assumed to reflect the true ancestral states. This procedure emulates “simple” or equally-weighted parsimony. In cases where multiple best reconstructions had the same probability (*i.e*., the same number of changes), the codon was not included in the analysis. *Up* and *pu* counts inferred using this procedure were compared to results from an iterative method of parsimony inference for 5 replicates each for initial MCU = 0.5, 0.7, and 0.9 for stationary (1x), variable *N*_e_ (1/3*N*_e_, 2*N*_e_) and variable mutation (2*u*/*v*, 0.5*u*/*v)* simulations scenarios (described below). Inferred numbers of changes were identical in all cases. Simple parsimony inference was chosen for this analysis because it has been employed extensively in Drosophila studies [*e.g*.08], [02], [14], [15], [06], [24], [25], [26], [16], [18].

The HKY85 substitution model [42] was also employed to infer ancestral codons using BASEML. This model incorporates unequal base composition and different transition and transversion rates. Four base composition parameters are estimated from the average base composition of the extant sequences. A transition/transversion rate ratio and branch lengths are estimated by maximizing their likelihoods over the data. The approach is similar to one employed by Galtier and Boursot [40] except that the BASEML model does not include a rate heterogeneity parameter and calculates substitution parameters separately for first, second, and third codon positions (in our simulations, only the third codon position is variable). It is important to note a difference in the parameterization of the HKY85 and GCpref codon substitution models; under HKY85, transversions to the same nucleotide are assigned the same rate (discussed below). The joint probabilities of codon reconstructions were calculated assuming independence among nucleotide reconstructions at different sites and a minimal evolution model was used to infer codon changes between nodes in the phylogeny. The probability of a given ancestral codon configuration was treated as the “count” of inferred substitutions on a given reconstruction. For example, consider a case where a configuration [G, A, G, G, G, G] for extant nodes [*m*, *s*, *t*, *y*, *e*, *o*] gave inferred ancestral configurations [G, G, G, G] and [A, G, G, G] for ancestral nodes [*ms*, *tyeo*, *ty*, *eo*] with probabilities 0.9 and 0.1, respectively. For this site, 0.9 G->A changes were recorded in the *s* lineage and 0.1 A->G changes were recorded in the *m* lineage. The latter change also requires either a G->A change in the *ms* lineage or an A->G change in the *tyeo* lineage but reconstructions were performed on an unrooted tree. This inference method will be referred to as “ML”. It is important to note that this method averages over possible ancestral reconstructions given their probabilities under the HKY85 substitution model and differs from ML implementations that treat the most probable reconstruction as pseudodata. The latter can yield strongly biased ancestral nucleotide frequencies for scenarios similar to those considered here [67].

MP and ML inferred codon bias changes were compared across 200–500 replicates for each scenario (numbers for each scenario are given in the figure legends). Mantel-Haentzel tests [68] were employed to detect consistent differences in ratios of MP and ML inferred counts of *up* and *pu* changes (summed across synonymous families). Wilcoxon ranked signs tests [68] were conducted to determine if differences between actual and inferred *d*_uppu_ were consistently smaller for MP or ML. We refer to a method as “more reliable” if inferred *up*/*pu* ratios differ between MP and ML (Mantel-Haentzel test, *P*<0.05) and *d*_uppu_ values are closer to actual values (Wilcoxon ranked signs test, *P*<0.05) for one of the methods. Computer programs for simulating sequence evolution and for analyses of BASEML results were written in the C computer language and are available upon request from HA.

#### Parsimony vs likelihood

**Figure 3:**
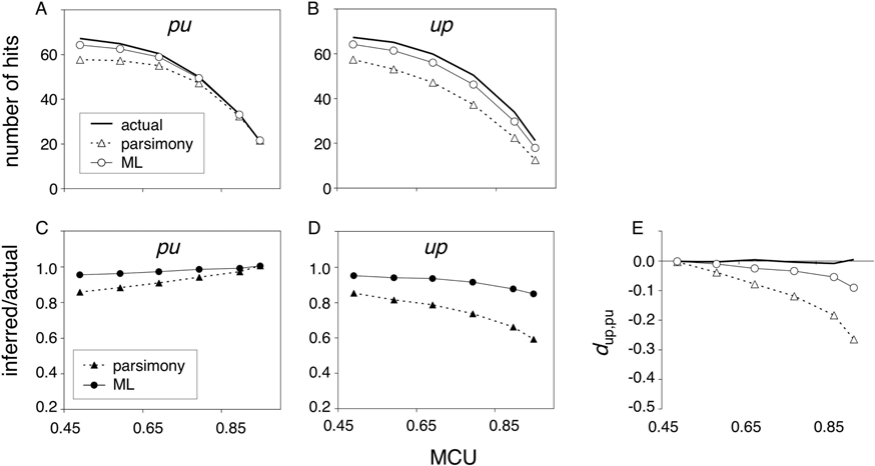
Inference of *pu* and *up* substitutions on the *m* lineage under equilibrium codon bias evolution. The numbers of hits and the ratios of inferred to actual hits reflect averages across 300 replicates of the 1x equilibrium scenario. X-axis scales apply to graphs in the same column. The legend in A applies to B and E and the legend in C applies to D. Reliability of *d*_up,pu_ inference was greater for ML than for MP for MCU≥0.6 (see text for criteria).

Figures 3A and B show the actual and inferred numbers of *pu* and *up* changes in the *m* lineage under a simulated equilibrium 1x tree. S->W changes are pooled in the *pu* class and W->S changes are pooled in the *up* class. Data for changes within preference classes (S->S and W->W) are not shown. Both MP and ML underestimate the numbers of changes and the underestimation is greater for MP inference, especially for *up* changes at high MCU. MP biases are similar to those described in a number of previous studies [19], [38], [39], [40], [41]. Figures 3C and D depict the ratio of inferred to actual numbers of changes and show that, for both methods, underestimation decreases with MCU for *pu* changes and increases with MCU for *up* changes. This results in a bias toward negative *d*_up,up_ (inflated inference of the ratio of *pu* to *up* fixations; Figure 3E) which increases with MCU for both MP and ML. The magnitude of the bias is considerably greater for parsimony. Under ML, inferred *pu*:*up* ratios are inflated by 4, 8, and 13% for MCU values of 0.5, 0.7, and 0.9, whereas for parsimony, *pu*:*up* is inflated by 16, 28, and 47% for the same MCU values. Even among closely related lineages at equilibrium base composition, biases in ancestral inference can generate patterns consistent with a genome-wide decline of MCU. Furthermore, the decrease in *d*_up,pu_ as a function of MCU mimics the expected trend following a reduction in *N*_e_ *s* (Figure 1B).

**Figure 4:**
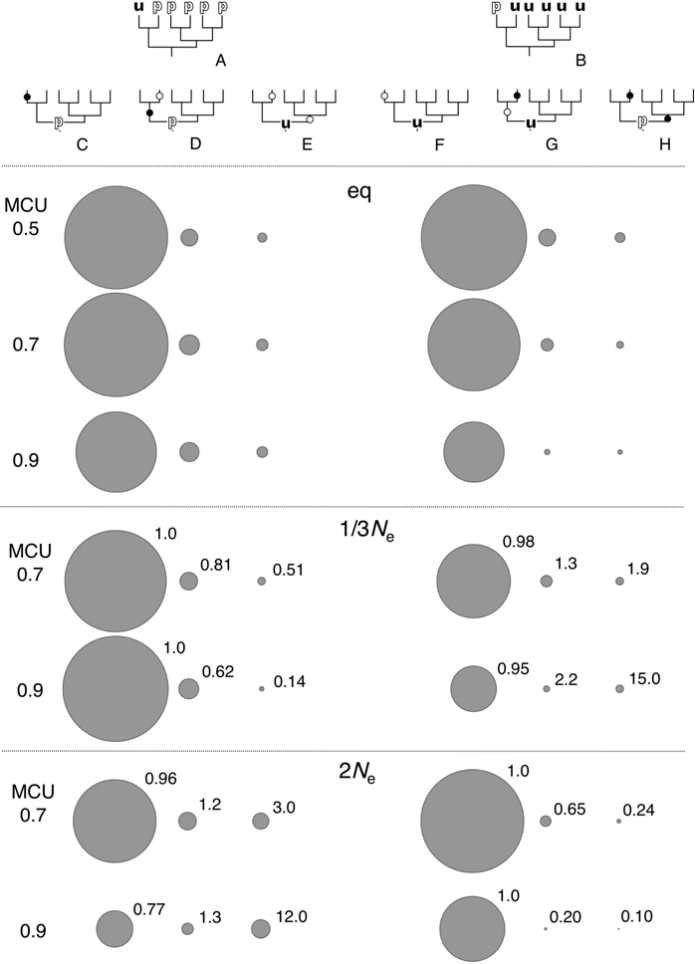
Ancestral codon configurations in simulations of codon bias evolution. Trees representing extant codon configurations consistent with single silent changes in the *m* lineage, ECC_uppppp (A) and ECC_puuuuu (E), are shown. The three most common ancestral codon configurations underlying these extant codon configurations are shown in B, C, and D for ECC_uppppp and in E, F, and G for ECC_puuuuu. Trees C and G reflect child/ancestor reverse changes and trees D and H show child/sib-ancestor parallel changes. The relative frequencies of ancestral codon configurations underlying ECC_uppppp and ECC_puuuuu in the codon bias simulations are shown as bubble plots beneath the trees. The sizes of the bubbles reflect the relative numbers of ancestral codon configurations in each class for three different MCU values. For the non-equilibrium scenarios (1/3*N*_e_ and 2*N*_e_), the proportion of each ancestral codon configuration among the extant configurations relative to the proportion under equilibrium codon bias evolution are given. The data are from Table 1.

Examples of extant and ancestral codon configurations illustrate some of the causes of these inference biases. Under neutrality (MCU = 0.5), the probabilities of *pu* and *up* fixations are equal. MP underestimation of the numbers of changes (Figure 3A and B) partly reflects the absence of inference in cases of multiple most parsimonious reconstructions. Greater inference bias as a function of MCU reflects a combination of differences in fixation probabilities and *per* locus mutation rates for *pu* and *up* changes. Figure 4B–D shows several ancestral codon configurations (ACCs) that could underlie an extant codon configuration (ECC) that MP would infer as a single *pu* change in the *m* lineage. The preference states of codons present in the extant nodes [*m*, *s*, *t*, *y*, *e*, and *o*] are [u, p, p, p, p, p] respectively (Figure 4A). Such an extant codon configuration will be abbreviated “ECC_uppppp” assuming all “p” states are identical. In the 1x equilibrium simulations, three ACCs underlie over 98% of ECC_uppppp's (Table 1). For MCU = 0.5, codons that underwent a single *pu* in the *m* lineage are the predominant scenario (94.6%), and double-hit codons with a *up* change in *s* and either a *pu* change in *ms* or a *up* change in *tyeo* underlie 3.5% of the configurations. In all cases, parsimony infers a single *up* change in the *m* lineage. For MCU = 0.5, the expected frequencies of each of the pairs of ACC types B and F, C and G, and D and H are equal since *pu* and *up* changes have the same rates [ECC_puuuuu and its ancestral codon configurations exchange p and u states at all nodes with those in ECC_uppppp (Figures 4E–H)]. Under the symmetric model of no selection and equal mutation rates, inference errors cancel. The scenarios depicted in Figures 4C and G will be referred to more generally as “child/ancestor reverse” changes (a child lineage is a direct descendent of an ancestral lineage; in these cases, *m* and *s* are the child lineages and *ms* is the ancestor) and those in Figures 4D and H will be referred to as “child/sib-ancestor parallel” changes (in these cases, *m* and *s* are child lineage and *tyeo* is the sibling to the ancestral *ms* lineage).

**Table 1:** Ancestral codon configurations underlying ECC_uppppp and ECC_puuuuu trees

Scenario^b^	MCU_i_ c	ECC_uppppp^a^				ECC_puuuuu^a^			
		total^d^	B	C	D	total	F	G	H
1x	0.5	13,688	12,951 (0.946)	364 (0.027)	110 (0.008)	14,156	13,441 (0.949)	369 (0.026)	123 (0.009)
	0.7	14,001	13,067 (0.933)	502 (0.036)	167 (0.012)	10,710	10,269 (0.959)	194 (0.018)	71 (0.007)
	0.9	8,744	7,934 (0.907)	486 (0.056)	142 (0.016)	4,618	4,498 (0.974)	41 (0.009)	9 (0.002)
1/3*N*_e_	0.5	13,768	13,059 (0.949)	358 (0.026)	109 (0.008)	13,626	12,939 (0.950)	322 (0.024)	113 (0.008)
	0.7	17,529	16,619 (0.948)	511 (0.029)	106 (0.006)	9,238	8,715 (0.943)	217 (0.023)	115 (0.012)
	0.9	19,039	18,034 (0.947)	653 (0.034)	44 (0.002)	3,623	3,368 (0.930)	72 (0.020)	104 (0.029)
2*N*_e_	0.5	13,725	13,046 (0.951)	315 (0.023)	112 (0.008)	13,929	13,219 (0.949)	329 (0.024)	127 (0.009)
	0.7	9,033	8,108 (0.898)	401 (0.044)	323 (0.036)	12,828	12,477 (0.973)	150 (0.012)	20 (0.002)
	0.9	2,307	1,609 (0.697)	166 (0.072)	438 (0.190)	5,056	4,992 (0.987)	9 (0.002)	1 (0.000)

a Extant codon configurations (ECC) from Figure 4.

b equilibrium (1x), decreasing (1/3*N*_e_), and increasing (2*N*_e_) codon bias simulations.

c Initial values of major codon usage (at the *mstyeo* node).

d Numbers of observations of each ECC and the numbers (proportions) of ancestral codon arrangements (ACC) underlying the ECC's (B, C, D and F, G, H correspond to ACC's shown in Figure 4). The numbers are pooled across 300 replicates and only trees with two different codon states among all nodes are included.

An important difference between the ECC_puuuuu and ECC_uppppp scenarios emerges when selection causes rate variation between mutation classes. For MCU = 0.9, ECC_uppppp's that result from multiply-hit codons with a *up* change in *s* (Figures 4C and D) increase to over 7% of the observations, whereas the fraction of ECC_puuuuu's resulting from a *pu* change in *s* at a multiply-hit codon (Figures 4G and H) *decreases* with MCU to less than 2% (Table 1). The asymmetry causes MP to overestimate the numbers of low probability (*pu*) changes, and underestimate the numbers of high probability (*up*) changes. This bias increases with the difference in the probabilities (*i.e*., with MCU). In addition, misinference of *up* changes in the *m* lineage following *pu* changes in *ms* (resulting in ECC_pupppp) as *pu* changes in *s* will further contribute to overestimation of *pu* in *m* and underestimation of *up* in *s*. Finally, MP inference attributes parallel changes in sibling lineages to single changes in the ancestral lineage. Among codons that experienced a *pu* change, the fraction that experienced parallel substitutions (another *pu* in a different lineage) decreases with MCU as selection reduces the probability of *pu* fixations. The proportion of multiple-*up* hit codons increases with MCU as selection elevates their fixation probabilities. This asymmetry contributes to parsimony underestimation of *d*_up,pu_.

#### Branch lengths

**Figure 5:**
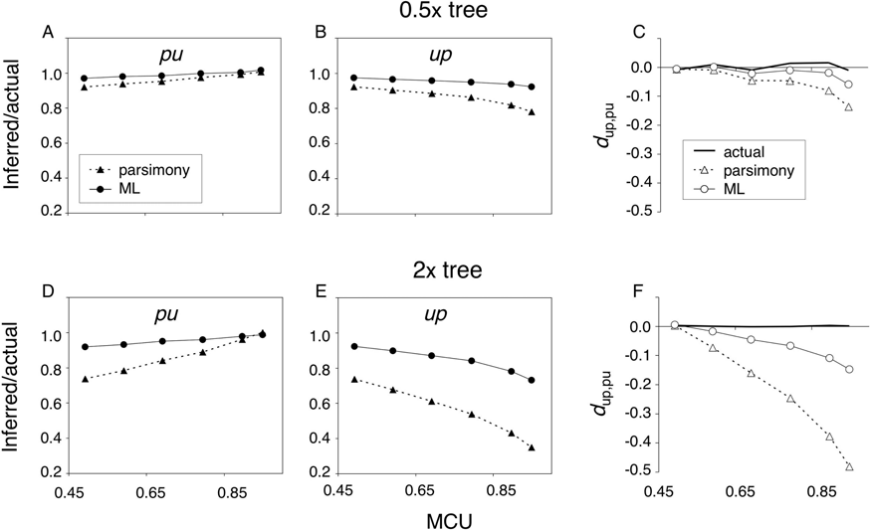
Reliability of ancestral codon bias inference under equilibrium evolution. A, B, and C show ratios of inferred to actual values and the *d*_up,pu_ for the 0.5× tree (averages among 500 replicates) and D, E, and F show values for the 2× tree (averages among 200 replicates). Actual and inferred numbers of hits are not shown. The legend in A applies to B, D, and E and the legend in C applies to F. X-axis scales are identical for graphs in the same column.

Reliability of ancestral inference is strongly dependent on distances among nodes [69]. Simulations of equilibrium codon bias were conducted for a tree with the same topology as shown in Figure 2 but with varying branch lengths. A 0.5× tree (half the 1× numbers of generations on all branches) and a 2× tree (double the 1× numbers of generations on all branches) were examined. Inference biases are smaller in the shorter tree (Figure 5C) and considerably greater in a 2× tree (Figure 5F). The MP bias in *d*_up,pu_ inference as a function of MCU in the 2× scenario is dramatic and reflects a striking underestimation of *up* changes (inferred values <50% of the actual numbers for MCU>0.8). These patterns reflect the increase in the proportions of multiply-hit codons with longer branch lengths (1.7, 5.7, and 16.7% of codons experienced >1 change since the *mstyeo* node in the 0.5×, 1×, and 2× scenarios, respectively for MCU = 0.7).

#### ML ancestral reconstruction bias

**Figure 6:**
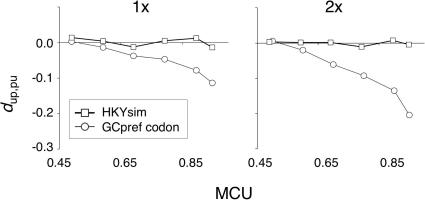
Reliability of ML inference for evolution under the GCpref codon and HKY85 models. *d*_up,pu_ values are shown for 4-fold synonymous codons for ML inference under the equilibrium GCpref codon model and for data simulated under the HKY85 substitution matrix. The latter matrix was set to give identical expected numbers of substitutions and equilibrium GC content for the two scenarios. The legend applies to both graphs and the y-axis scales are identical in the two graphs. Note that *d*_up,pu_ inference biases are larger for 4-fold redundant codons than for 2-fold redundant codons under the GCpref codon model. Data are averaged across 300 replicates.

ML inference, though more reliable than MP, also shows a bias toward negative *d*_up,pu_ with MCU (especially in the 2× tree; Figure 5F). Under the HKY85 model, four base composition parameters [***π***  =  (*π*_A_, *π*_T_, *π*_C_, *π*_G_)] are estimated from extant sequences and the transition/transversion rate ratio (*κ*) is estimated from patterns of sequence divergence. Because the parameters are calculated separately for the three codon positions and because the simulated scenario is one of equilibrium base composition, ancestral inference should be more accurate than MP. Differences in the parameterizations of the HKY85 and GCpref codon models appear to underlie the observed ML biases. Under HKY85, transversion changes to the same nucleotide occur at the same rate. For example, C->A and T->A occur at rate *π*_A_ and A->C and G->C occur at rate *π*_C_. Under the GCpref codon model, transversions to the same nucleotide consist of one mutation affecting fitness (C->A is unpreferred and A->C is preferred) and one neutral mutation (within a fitness class: T->A and G->C). If a single rate is estimated for transversions to A, the C->A (unpreferred) rate will be overestimated and the T->A (neutral) rate will be underestimated. For transversions to C, the A->C rate will be underestimated and the G->C rate will be overestimated. Overall, the rates of preferred and unpreferred transversions will be underestimated and overestimated, respectively, and this error will increase with MCU. This leads to underestimation of *d*_up,pu_ with MCU, a bias similar to MP. ML inference for data simulated under the exact parameterization of the HKY85 model showed no bias in *d*_up,pu_, even under strong base composition bias in a 2× tree (Figure 6). Seemingly minor deviations from model assumptions can lead to considerable biases for relatively highly biased genes and/or long branches.

#### Changes in selection intensity

Constancy of parameters governing molecular evolution may often be violated in Drosophila among closely related lineages [08], [02], [20], [21], [06], [70], [15], [24], [25], [26], [16], [35], [37] as well as among distantly related taxa [71], [22], [23], [27]. Ancestral inference under fluctuating codon bias is examined below.

Two scenarios of changes in selection intensity were examined: a three-fold decrease in *N*_e_ (1/3*N*_e_) and a two-fold increase in *N*_e_ (2*N*_e_). Parameter changes were invoked at the *mstyeo* node and substitution probabilities differing from those in the *mstyeo* lineage were employed for evolution within the *D. melanogaster* subgroup. These probabilities were kept constant within the subgroup. However, because base composition changes directionally during the course of the simulation, *per* locus mutation rates are not constant.

**Figure 7:**
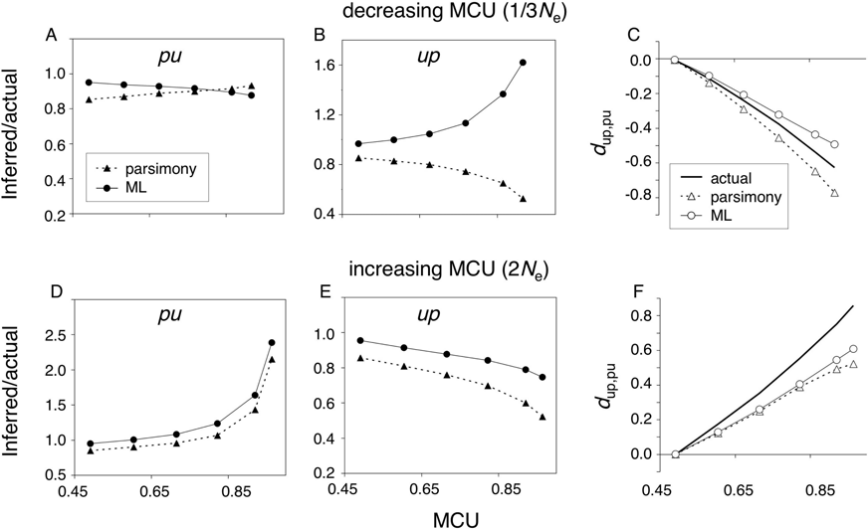
Reliability of ancestral codon bias inference under non-equilibrium evolution: variable selection intensity. The legend in A applies to B, D, and E. The legend in C also applies to F. X-axis scales are identical for graphs in the same column. Note that the MCU values reflect values at the *m* node and differ from ancestral values. For the 1/3*N*_e_ scenario, reliability of *d*_up,pu_ inference was greater for ML than for MP for 0.6≤MCU≤0.8. For 2*N*_e_, ML was more reliable for MCU≥0.8 (see text for criteria). Data are averaged across 300 replicates.

For the 1/3*N*_e_ scenario (GC content decline), MP and ML show contrasting biases in *d*_up,pu_ inference (Figures 7A to C). Parsimony biases are similar to the equilibrium case; both *pu* and *up* changes are underestimated, but the magnitude of *pu* underestimation *decreases* with MCU whereas *up* underestimation *increases* as a function of codon bias. *d*_up,pu_ is biased downward and the bias increases with MCU in a manner similar to the equilibrium case. Because the actual *d*_up,pu_ is negative (Figure 1B), MP exaggerates reductions of MCU.

Examination of extant and ancestral codon configurations reveals differences in the causes of similar MP reconstruction biases under equilibrium and decreasing codon bias. Under reduced *N*_e_, the ratio of fixation probabilities of *pu* and *up* mutations decreases less as a function of MCU than under stationarity. Table 1 and Figure 4 show that, among ECC_uppppp's, the frequencies of multiply-hit codons do not increase with MCU as in the equilibrium case. However, increases in the *numbers* of ECC_uppppp's with MCU (in contrast to decreases under equilibrium) lead to considerable numbers of misinferred *up* changes at high MCU. For ECC_puuuuu, the frequency of multiply-hit codons increases with MCU rather than decreasing as in the equilibrium scenario. The prevalence of ECC_uppppp results in *d*_up,pu_ inference biases similar to the equilibrium case.

ML inference of *pu* and *up* changes under relaxed selection differs considerably from MP results. *pu* underestimation increases and *up* changes are *overestimated* with increasing *N*_e_ *s*. This causes overestimation of *d*_up,pu_ as a function of MCU (bringing *d*_up,pu_ closer to zero; Figure 7C). The bias in the opposite direction compared to ML inference under equilibrium (Figure 3E) is a consequence of the method for estimating base composition parameters. The lineages examined are relatively short compared to the time required to reach equilibrium. The base composition of extant sequences reflects parameter values (*N*_e_ *s* and *u*/*v*) from the ancestral *mstyeo* lineage rather than values on the lineages on which fixations are inferred. For a given ECC, ancestral reconstructions are assigned probabilities according to equilibrium expectations given the base composition of the extant sequences; rate parameters are biased upward for *up* changes and downward for *pu* changes. *d*_up,pu_ inference is biased toward positive values because parallel *up* changes are assigned inflated probabilities and parallel *pu* changes are given underestimated probabilities. For ECC_uppppp, the proportions of codons with multiple *up* changes are considerably smaller than under equilibrium (roughly 50 and 14% for MCU = 0.7 and 0.9, respectively; Figure 4; Table 1) and ML overestimates this proportion. The larger *numbers* of codons with *pu* changes leads to considerable *up* overestimation (Figure 7B). For ECC_puuuuu, the proportions of codons with multiple *pu* changes are much *larger* than under equilibrium (roughly 2× and 15× for MCU = 0.7 and 0.9, respectively) and ML underestimates their probabilities.

Under the 2*N*_e_ scenario (increasing GC), both MP and ML underestimate *d*_up,pu_ as a function of MCU (Figures 7D–F). Actual *d*_up,pu_ values are positive (Figures 1C and D) and both methods bias the statistic toward zero. Because base composition in extant sequences reflects parameter values prior to the increase in *N*_e_ (*i.e*., lower GC content), HKY85 underestimates *up* rates and overestimates *pu* rates. Ancestral unpreferred sites have a much larger probability of undergoing *up* changes in multiple lineages than under equilibrium (Figure 4; Table 1); for MCU = 0.9, over 30% of ECC_uppppp observations reflect multiply-hit codons. Both ML and MP underestimation of *up* is larger than in the equilibrium case, but ML underestimation is less severe because multiple-*up* hit scenarios are assigned some probability. For ECC_puuuuu, multiple-hit scenarios are less common than under equilibrium (Figure 4; Table 1). Thus, MP shows less underestimation of *pu* as a function of MCU than in the equilibrium case, but ML overestimates *pu* changes. In contrast to the 1/3*N*_e_ scenario, the numbers of ECC_puuuuu observations are larger than the numbers of ECC_uppppp's for high MCU values. The magnitude of the resulting *pu* overestimation is substantial for both MP and ML (Figure 7D). Under this particular departure from the assumptions of both methods, ML and MP biases in *d*_up,pu_ inference are similar across a wide range of MCU.

#### Changes in mutation bias

**Figure 8:**
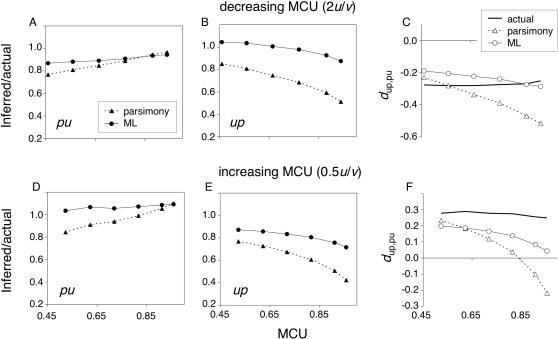
Reliability of ancestral codon bias inference under non-equilibrium evolution: variable mutation. The legend in A applies to B, D, and E. The legend in C applies to F. X-axis scales are identical for graphs in the same column. Note that MCU values are given for the *m* node and have shifted from ancestral values. For the 2*u/v* scenario, reliability of *d*_up,pu_ inference was greater for ML than for MP for MCU≥0.8, but MP was more reliable for MCU≤0.6. For 0.5*u/v*, *d*_up,pu_ was more reliably inferred by ML than by MP for MCU≥0.7, but MP was more reliable for MCU = 0.5. Data are averaged across 300 replicates.

Ancestral inference were also examined for non-stationary codon bias evolution following changes in mutation rates. For these simulations, expected *up*:*pu* ratios of 0.5 and 2.0 were achieved by doubling either *u* (*per* site rate of S->W mutations) or *v* (*per* site rate of W->S mutations) relative to their values in the stationary 1× simulations. The scenarios will be referred to as 2*u*/*v* and 0.5*u*/*v*, for doublings of *u* and *v*, respectively. Within-fitness class mutation rates, W->W and S->S, were not changed. Mutation rate changes were implemented at the *mstyeo* node and all parameters were kept constant following the change. Expected *pu*:*up* fixation ratios are equal to the new *u*:*v* ratios immediately following a change in the mutation rate, but the fixation ratios gradually approach the equilibrium value of one (rates of approach to equilibrium are positive functions of initial MCU). Thus, the actual excesses of *up* or *pu* changes on the *m* lineage are smaller than two-fold and decrease with MCU (Figures 8C and F).

For the 2*u*/*v* scenario of mutation-driven reductions in MCU, parsimony underestimates decreases in codon bias for low MCU genes, but overestimates codon bias changes for higher MCU (Figure 8C). In the absence of selection (MCU ≈ 0.5), parsimony underestimation is greater for the more common *pu* changes than for *up* changes. Increasing underestimation of *d*_up,pu_ as a function of MCU is similar to the pattern observed under equilibrium (Figure 3E). As selection increases the ratio of *up*:*pu* fixation probabilities, parsimony misinference at multiple *up*-hit codons leads to underestimation of *up* changes.

ML underestimates mutation-driven decreases in codon bias for low and intermediate MCU. Because parameters are estimated from sequences that have not reached equilibrium base composition, rates of *up* and *pu* are over- and under-estimated, respectively. This bias in parameter estimation leads to error in the probabilities of joint reconstructions. Decreasing underestimation of *d*_up,pu_ with MCU (the decline of codon bias is overestimated at very high MCU) probably reflects differences between the HKY85 and GC codon pref models (rates are estimated for pooled classes of neutral and non-neutral mutations; see above). With increasing MCU, the latter bias compensates, then over-compensates, for the bias introduced by non-stationary base composition.

Inference biases were considerable for mutation-driven increases in codon bias. In the 0.5*u*/*v* scenario, both selection and mutation elevate ratios of *per* site *up*:*pu* substitutions. Parsimony misinference at multiple-*up* hit codons increases dramatically as a function of MCU (Figure 8E) and results in large underestimation of *d*_up,pu_ (Figure 8F). For initial MCU = 0.95, parsimony infers *d*_up,pu_ = −0.22 where the actual value is 0.25.

ML biases were similar in direction, but much smaller in magnitude, than parsimony biases for the 0.5*u*/*v* scenario. Inference biases reflect under- and over-estimation of *up* and *pu* rates because base composition parameters are estimated from sequences that are far from equilibrium. Fitting data generated under GCpref codon to the HKY85 model exacerbates these biases, leading to greater differences between inferred and actual *d*_up,pu_ than for ML in the 2*u*/*v* scenario.

#### Lineage-specific departures from equilibrium

The non-stationary scenarios considered above have employed a single parameter change at the *mstyeo* node and similar departures from equilibrium among lineages. A large number of scenarios of lineage-specific departures from equilibrium are possible, but some general results can be obtained from consideration of a set of scenarios based on findings in the *D. melanogaster* subgroup.

Data from nineteen loci are consistent with reductions in codon bias in the *m*, *y*, *o*, and *eo* lineages, and increases in codon bias in the *t* and *ty* lineages [35]. For these genes, patterns on the *s* and *e* lineages appear to be consistent with equilibrium MCU. Changes in codon bias were assigned accordingly in the simulation. For simplicity, all reductions in codon bias were modeled as 1/3*N*_e_ and all increases were modeled as 2*N*_e_. Because changes in the ancestral *ms* and *tyeo* lineages are unknown, all combinations of increases (2*N*_e_), decreases (1/3*N*_e_), and stationarity for these two lineages were employed (nine scenarios). Parameters were changed at the ancestral node of a lineage and were held constant within the lineage.

**Figure 9:**
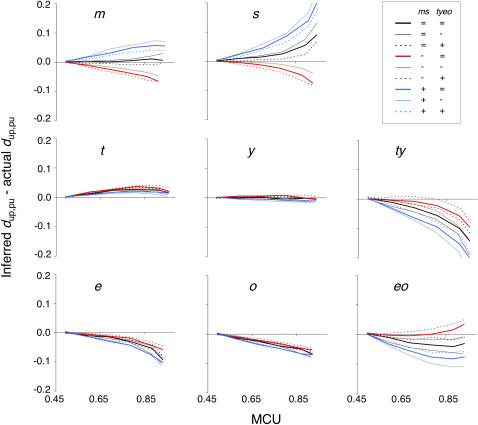
Reliability of ML codon bias inference under lineage-specific non-stationarity. Differences between inferred and actual *d*_up,pu_ values are plotted as a function of MCU for each lineage (averages across 300 simulations are plotted). The legend applies to all graphs. X-axis scales apply to all graphs in the same column. Y-axis scales are identical among all graphs. The lineage-specific scenarios are as follows: stationary MCU (st) in <l>s and *e*, decreasing MCU (1/3*N*_e_) in *m, y, o,* and *eo*, and increasing MCU (2*N*_e_) in *t* and *ty*. Scenarios were varied in the ancestral *ms* and *tyeo* lineages. For the *ms* lineage: black (st), red (1/3*N*_e_), blue (2*N*_e_). For the *tyeo* lineage: thick (st), thin (1/3*N*_e_), dotted (2*N*_e_).

To allow comparisons between equilibrium and lineage-specific non-equilibrium simulations, *differences* between inferred and actual *d*_up,pu_ are plotted for each lineage in Figure 9 and Figure S1. Given the phylogenetic distances in the *D. melanogaster* subgroup, *d*_up,pu_ inference biases under the mixed non-equilibrium simulation can be understood, to a large extent, by considering extant codon configurations consistent with both a single change or changes in two lineages (ECC_SD for extant codon configurations consistent with single or double hits). Because the *y* lineage is decreasing in bias and the ancestral *ty* lineage is increasing in MCU, *up* in *ty* followed by *pu* in *y* has an elevated occurrence relative to the 1× stationary case. *d*_up,pu_ under ML inference is elevated in *t* and *y*, and reduced in *ty*. Because *y* and *eo* are both losing codon bias, parallel *pu* changes in these lineages are more common, but such a scenario does not result in an ECC_SD. Relative to the equilibrium case, a decline of codon bias in the *m* lineage elevates the probability of *up* changes in *ms* followed by *pu* reversals in *m*. Because ML underestimates this probability, *d*_up,pu_ is elevated in both *m* and *s*.

Rate heterogeneity in ancestral lineages can have a strong impact on inference in their descendent lineages. If codon bias is increasing in the *ms* lineage (blue lines in Figure 9), then *up* in *ms*/*pu* in *m* reversals become more common. Such changes elevate *d*_up,pu_ for both the *m* and *s* lineages. In addition, increased frequencies of parallel *up* changes in the *ms* and *ty* lineages decrease *d*_up,pu_ in both *ty* and in *eo*. The length of the *ms* lineage contributes to substantial effects of these double-hit scenarios on *d*_up,pu_ inference. Decreases in MCU on the *ms* lineage (red lines in Figure 9) have the opposite effect on *d*_up,pu_ inference. Reverse changes, *pu* in *ms*/*up* in *s,* cause decreased *d*_up,pu_ inference in *m* and *s*, and parallel *pu* changes in *ms* and *eo* result in elevated *d*_up,pu_ in *ty* and *eo* (relative to equilibrium in *ms*). Note that *d*_up,pu_ inference on the *t*, *y*, *e*, and *o* lineages is relatively insensitive to departures from stationarity in *ms* and *tyeo* (Figure 9).

Non-stationary evolution on the ancestral *tyeo* lineage has a smaller impact on *d*_up,pu_ inference than departures from equilibrium on the longer *ms* lineage. Increasing MCU on the *tyeo* lineage enhances the probability of *up* in *tyeo*/*pu* in *eo* reversals. This results in elevated *d*_up,pu_ in both *eo* and *ty*. Higher occurrences of parallel *up* changes in *s* and *tyeo* result in decreased *d*_up,pu_ in both *s* and *m*. Decreasing codon bias in *tyeo* has the opposite effects on *d*_up,pu_ inference in these lineages. The elevated occurrence of *pu* in *tyeo*/*up* in *ty* reversals decreases *d*_up,pu_ in *ty* and *eo* and parallel *pu* changes in *tyeo* and *m* leads to elevated *d*_up,pu_ for the *s* and *m* lineages.

MP performance can be understood by considering the same sets of ECC_SD's. Inference biases (relative to the stationary 1× case) for the mixed non-equilibrium simulations are similar in direction, but generally greater in magnitude, to those under ML. Figure S1 shows considerable MP underestimation of *d*_up,pu_, even at intermediate MCU, for the *m*, *s*, *e*, *o*, and *eo* lineages for most of these scenarios.

These results suggest that ML generally allows more reliable inference of ancestral codon usage than MP for lineages within the *D. melanogaster* subgroup. In addition, inference biases do not appear to have contributed substantially to the conclusion of frequent codon bias fluctuations within the *D. melanogaster* subgroup [35]. In particular, biases in ML *d*_up,pu_ inference for the *t*, *y*, *e*, and *o* lineages appear to be relatively small if the magnitude and direction of departures from equilibrium in our simulations are correct. Inference biases on the *s*, *ty* and *eo* lineages, however, can be considerable if codon bias has increased on the *ms* lineage. The sensitivity of inference on the *s* lineage to processes occurring in ancestral lineages appears to result from the combination of a strong departure from equilibrium on a sibling lineage, a long parent lineage, and the lack of an outgroup for the *ms* clade. It is important to note that longer lineages or greater departures from equilibrium could elevate *d*_up,pu_ biases from those shown in Figure 9. Other factors that affect ancestral inference (*i.e*., the locations of lineages in the phylogeny and species sampling) are discussed in Results S1 and S2.

## Discussion

### Reliability of ancestral codon usage inference

The accuracy of ancestral inference, even among relatively closely related species, has a complex dependence on branch lengths, the location of lineages within a phylogeny, rate heterogeneity among mutation classes (levels of base composition bias) and among lineages (changes in base composition). Parsimony is susceptible to errors in ancestral reconstruction when base composition is biased [19], [38], [39], [40], [41]. For lineages at stationary base composition, inference biases can yield patterns mimicking departures from equilibrium codon bias toward a loss of the common state (excess of unpreferred substitutions); the increase in this bias with the degree of base composition skew can lead to false inference of reductions in selection intensity for codon bias.

For the scenarios considered in this study, maximum likelihood implementation of the HKY85 model is generally (often considerably) less biased than MP. Estimation of separate substitution rates for classes of nucleotide changes is desirable when base composition is skewed. However, departures from steady-state can cause strong biases in ancestral reconstruction among closely related species. The long lag-time between parameter changes and the achievement of a new equilibrium base composition is problematic for models that infer substitution rates from the base composition of extant sequences. Because the time-scale on which parameters vary appears to often be shorter than the time scale over which equilibrium is achieved [35], [37], then inference using stationary models must be treated with caution.

The direction and magnitude of departures from equilibrium as well as the branch lengths of a given lineage, its sibling lineage, direct ancestral lineages, and the sibling lineages to its direct ancestors can have a strong impact on the reliability of ancestral inference. For example, child/ancestor reversals will be prevalent if departures from equilibrium are in opposing directions in branches with direct descendent relationships. Parallel changes will be common when departures from equilibrium are in the same direction in sibling lineages or in a lineage and the sibling lineage of a direct ancestor. Bursts of adaptive codon bias evolution on multiple lineages can substantially reduce the reliability of ancestral inference.

Among the lineages in our simulated data, ancestral inference is generally less biased in the *t*, *y*, *e*, and *o* lineages than in the *m*, *s* and *ty*, *eo* lineages. The greater accuracy of inference for terminal lineages in the *tyeo* clade results, in part, from shorter parental lineages than for the *m* and *s* lineages. In addition, for the *t, y, e,* and *o* lineages, data from the outgroup *ms* clade often eliminates the possibility of single-hit ancestral configurations consistent with child/sib-parent parallel changes. For these lineages, inference biases can be predicted given sequence data from species within the subgroup whereas biases for the *m, s*, *ty* and *eo* lineages are strongly dependent on unknown processes in the ancestral *ms* and *tyeo* lineages (Figure 9).

### Improving inference methods for ancestral codon usage

Reliability of ancestral reconstructions is compromised by incorrect trees, errors in sequence alignment, incorrect substitution models, and insufficient data to estimate parameters of the substitution model. The analyses above assumed knowledge of the correct tree topology, no alignment errors, and sufficient data for ML parameter estimation. This study focused on the effects of incorrect substitution models.

Ancestral reconstructions can be improved both by data selection and by model improvements. Blanchette *et al*. [72] found that a “star-like” phylogeny allows reliable inference of ancestral mammal sequences given stationary evolution and sufficient sampling of extant taxa. Branch lengths are critical determinants of the accuracy of ancestral inference and errors can be minimized by choosing data from closely related species (with short ancestral lineages). However, even for the low levels of divergence examined in this study, departures from substitution model assumptions led to biases in ancestral reconstructions for high MCU genes (for MP) and for departures from steady-state (for MP and ML). These effects can be minimized by restricting data to moderately biased genes (MCU<0.8). However, biases were clearly detectable for intermediate MCU genes in a number of examined scenarios, and longer lineages or greater fluctuations in base composition than those studied here can inflate these biases. For example, our simulations assumed homogeneity of parameters within genes. A number of findings support within-gene heterogeneity in *N*_e_ *s* at silent sites [73], [74], [75], [20], [21], [76], [77], [17], [78] which could elevate inference biases because rate parameters at variable sites will be underestimated.

A number of improvements in ancestral inference methods could enhance comparisons among closely related species. Uncertainties in both tree topologies and parameter estimates have been incorporated in Bayesian approaches to ancestral state reconstruction [79], [80], [67], [81]. Rate heterogeneity parameters are included in Galtier and Boursot's [40] ML method. The equilibrium assumption appears to be a critical limitation in the methods employed here and Yang and Roberts [82] and Galtier and Guoy [83] have incorporated parameters for fluctuating base composition in methods to infer ancestral GC content (these implementations do not calculate posterior probabilities of ancestral states).

Modifications to the simple parsimony approach could also improve inference of ancestral codon usage. The MP method employed above eliminated data for codons at which multiple reconstructions give the least number of changes in the gene tree. Such data could be included by allocating equal probabilities among most parsimonious reconstructions. In addition, classes of mutations can be assigned “weights” to account for asymmetric substitution rates [84].

Key considerations for substitution models may vary considerably among taxa and genetic distances. Neighboring base effects play a strong role in mammalian genomes and transition/transversion rate ratios can be large in mtDNA evolution [85]. For evolutionary studies of coding regions within the *D. melanogaster* subgroup, a codon substitution model that accommodates both heterogeneity in rates among sites and fluctuations in base composition may be appropriate. Nielsen et al. [86] have recently developed a method that allows fluctuations in codon bias within a gene tree and Arndt [33] and Hernandez and co-workers [87] have proposed methods that account for non-stationarity and neighboring base effects. However, such models may entail trade-offs; increases in the amount of data required to estimate a larger number of parameters may preclude analyses of gene- and lineage-specific evolution except for large genes and/or long lineages [83]. The average inferred numbers of *pu* and *up* per gene per lineage are 8.0 and 5.7, respectively, within the *D. melanogaster* subgroup for the nineteen genes examined in [35].

Finally, the data simulated for this study were generated using an instantaneous substitution model and did not consider variation within species. For several lineages in the *D. melanogaster* subgroup, the most recent common ancestor for within-species alleles may lie close to ancestral nodes. Contrasts of the numbers and the site frequency spectra of newly arisen mutations within populations and among closely related species [88], [89], [08], [10], [90], [13] can be powerful approaches to identify the roles of weak selection and adaptive evolution for different functional classes of mutations [09], [91]. Such methods can determine the causes of codon bias and protein evolution as well as mechanisms of regulatory region and intron evolution [*e.g*., 92]. Studies of the reliability of ancestral inference for polymorphism/divergence data [*e.g*., 93] will be necessary to determine the robustness of such analyses.

## Supporting Information

Methods S1 The major codon preference model.
(0.04 MB DOC)

Figure S1 Reliability of parsimony codon bias inference under lineage-specific non-stationarity. Differences between inferred and actual *d* ^up,pu^ values are plotted as a function of MCU for each lineage (averages across 300 simulations are plotted). The legend applies to all graphs. X-axis scales apply to all graphs in the same column. Y-axis scales are identical among all graphs and are identical to those in Figure 9 to allow comparisons between methods. The lineage-specific scenarios are identical to those in Figure 9: stationary MCU (st) in *s* and *e*, decreasing MCU (1/3*N* ^e^) in *m, y, o,* and *eo*, and increasing MCU (2*N* ^e^) in *t* and *ty*. Scenarios were varied in the ancestral *ms* and *tyeo* lineages. For the ms lineage: black (st), red (1/3*N* ^e^), blue (2*N* ^e^). For the *tyeo* lineage: thick (st), thin (1/3*N* ^e^), dotted (2*N* ^e^).
(16.60 MB TIF)

Results S1 Lineage-dependent ancestral reconstruction biases.
(1.68 MB DOC)

Results S2 Species composition and ancestral reconstruction biases.
(0.64 MB DOC)
