# Supplementary material for: Ancestral Inference and the Study of Codon Bias Evolution: Implications for Molecular Evolutionary Analyses of the Drosophila melanogaster Subgroup
Source: PLoS One. 2007 Oct 24;2(10):e1065. doi: 10.1371/journal.pone.0001065 (PMC2020436; doi:10.1371/journal.pone.0001065)
Supplement: Methods S1 — The major codon preference model. (0.04 MB DOC) [file pone.0001065.s001.doc]

Supporting Information for Akashi et al. “Ancestral inference and the study of codon bias….”

**Methods S1 The major codon preference model**

Evolutionary dynamics under major codon preference have been studied by Li [S1] and Bulmer [S2]. The following assumes the simplest form of the model for a binary character (*i.e*., two-fold redundant codon) as described in the text. Fixation probabilities of *pu* and *up* mutations can be calculated using Kimura’s [S3] expressions assuming semi-dominant fitness effects. Consider a “locus” consisting of *l* sites occupied by either the A_1_ or A_2_ state. The rates of *pu* and *up* substitution are the products of their mutation rates, 2*Nlqu* and 2*Nl*(1-*q*)*v*, and their fixation probabilities, *P*_pu_ and *P*_up_, respectively. If *N*_e_*u* << 1and *N*_e_*v* << 1, levels of polymorphism will be negligible and a frequency of A_1_ states, *q*, at equilibrium can be calculated by setting *per* locus substitution rates equal for *pu* and *up* changes. Assuming homogeneity of *N*_e_*s* and *u*/*v* across sites and over time,

*q* ≈ (1) [S1, S2].

Note that both *q* and MCU refer to the frequency of major codons at a locus. Equality of expected *per* locus *pu* and *up* substitution rates requires that the ratio of their fixation probabilities equal the reciprocal of their *per* locus mutation rates,

(2).

The equilibrium MCU model described above assumes constant *u*/*v* and *N*_e_*s* over a large number of generations. However, MCU is highly sensitive to parameter fluctuations. Consider a locus at an equilibrium usage of A_1_. If *N*_e_*s* increases, the fixation probabilities of *pu* and *up* mutations decrease and increase, respectively, leading to an excess of *up* fixations and an elevation of *q*. As *q* increases, the *per* locus *up* and *pu* mutation rates decrease and increase, respectively, until the ratio of these rates is equal to the reciprocal of their fixation probabilities. Equilibrium MCU is approached on a time scale on the order of the reciprocal of *per* site mutation rate. For example, for a gene with initial MCU=0.7, new equilibrium MCU values will be 0.844 and 0.572 under a 2-fold increase and decrease to 1/3 of initial *N*_e_*s*, respectively (for *u*/*v*=1). The numbers of generations required for MCU to travel half the distance to these equilibrium values are approximately 0.28 and 0.33 times the reciprocal of the mutation rate for the increase and decrease in MCU, respectively.

**REFERENCES**

S1. Li WH (1987) Models of nearly neutral mutations with particular implications for nonrandom usage of synonymous codons. J Mol Evol 24: 337-345.

S2. Bulmer M (1991) The selection-mutation-drift theory of synonymous codon usage. Genetics 129: 897-907.

S3. Kimura M (1962) On the probability of fixation of mutant genes in a population. Genetics 47: 713-719.
