# Supplementary figures and images for: Ancestral Inference and the Study of Codon Bias Evolution: Implications for Molecular Evolutionary Analyses of the Drosophila melanogaster Subgroup
Source: PLoS One. 2007 Oct 24;2(10):e1065. doi: 10.1371/journal.pone.0001065 (PMC2020436; doi:10.1371/journal.pone.0001065)

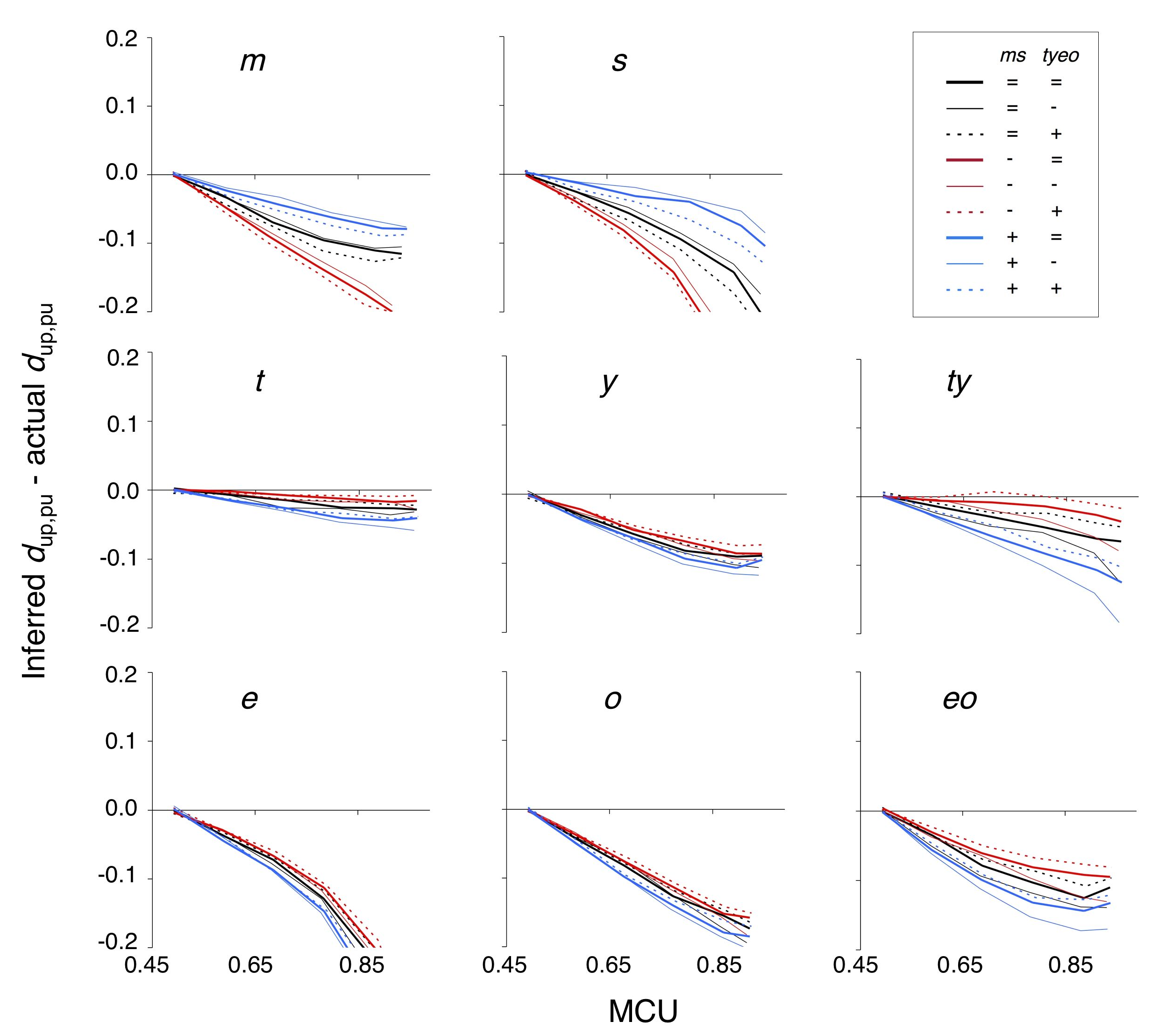

Supplement: Figure S1 — Reliability of parsimony codon bias inference under lineage-specific non-stationarity. Differences between inferred and actual d up,pu values are plotted as a function of MCU for each lineage (averages across 300 simulations are plotted). The legend applies to all graphs. X-axis scales apply to all graphs in the same column. Y-axis scales are identical among all graphs and are identical to those in Figure 9 to allow comparisons between methods. The lineage-specific scenarios are identical to those in Figure 9: stationary MCU (st) in s and e, decreasing MCU (1/3N e) in m, y, o, and eo, and increasing MCU (2N e) in t and ty. Scenarios were varied in the ancestral ms and tyeo lineages. For the ms lineage: black (st), red (1/3N e), blue (2N e). For the tyeo lineage: thick (st), thin (1/3N e), dotted (2N e). (16.60 MB TIF) [file pone.0001065.s002.tif]
