# Supplementary material for: Ancestral Inference and the Study of Codon Bias Evolution: Implications for Molecular Evolutionary Analyses of the Drosophila melanogaster Subgroup
Source: PLoS One. 2007 Oct 24;2(10):e1065. doi: 10.1371/journal.pone.0001065 (PMC2020436; doi:10.1371/journal.pone.0001065)
Supplement: Results S1 — Lineage-dependent ancestral reconstruction biases. (1.68 MB DOC) [file pone.0001065.s003.doc]

Supporting Information for Akashi et al. “Ancestral inference and the study of codon bias….”

**Results S1 Lineage-dependent ancestral reconstruction biases**

For simulations that employ common parameters throughout the gene tree, the eight lineages on which changes are inferred fall into three categories: [*m*, *s*], [*ty*, *eo*], and [*t*, *y*, *e*, *o*]. Expected within-lineage evolution and the locations and processes occurring in neighboring lineages are equivalent within each of the categories. Inference biases differences among lineages can be understood by considering extant codon configurations consistent with both a single change or changes in two lineages (ECC_SD’s). Reversals in a lineage and a direct ancestral lineage (a parental lineage or a parent of a parent, *etc*) and parallel changes in a lineage and either its sibling or the sibling of a direct ancestral lineage can cause differences in the reliability of *d*_up,pu_ inference for the *m*, *t*, and *ty* lineages. Although reconstruction accuracy is similar among the lineages under stationary base composition, among-lineage differences in *d*_up,pu_ bias become more pronounced under departures from equilibrium. Reliability of reconstructions among lineages depends on the branch lengths and locations of neighboring lineages as well as processes within the lineages.

***m* lineage:**  Ancestral inference at the *ms* node can be complicated by a large number of ECC_SD possibilities. Reversals in *m* and *ms* are consistent with a single change in *s*. Inference on *m* is also affected by both types of reversals in *s* and *ms*. Parallel changes in the *m* and *s* lineages are consistent with single changes in the ancestral *ms* lineage. Parallel changes in *m* and *tyeo* are consistent with single changes in the *s* lineage and parallel changes in *s* and *tyeo* are consistent with single changes on the *m* lineage. The relative probabilities of single- vs. multiple-hit codons depends on both branch lengths and departures from equilibrium on the *m*, *s*, *ms*, and *tyeo* branches.

***t* lineage:** The *t* lineage has a shorter parent lineage (*ty*) than the *m* lineage and is surrounded by a larger number of nearby nodes. Inferring codon bias evolution on the *t* lineage is complicated by fewer ECC_SD possibilities than the *m* lineage. Parallel changes on the *t* and *y* lineages are consistent with a single change in the ancestral *ty* lineage. In addition, reverse changes on the *t* and *ty* lineages or on the *y* and *ty* lineages will affect inference of changes on the *t* lineage. However, extant codon configurations resulting from parallel changes on either the *t* and *eo* or the *y* and *eo* lineages are not consistent with single-hit scenarios. In such cases, data from the outgroup *ms* clade enhances ancestral inference in the *tyeo* clade. Similarly, reverse changes in the *t* and *tyeo* or *y* and *tyeo* lineages do not result in ECC_SD’s (unless an additional change occurs in the *ms* clade).

Single *pu* and *up* substitutions on the *t* lineage result in ECC_ppuppp and ECC_uupuuu, respectively. Because fewer double-hit codons are consistent with these configurations, the probabilities that these configurations reflect single changes are higher than for equivalent configurations in the *m* lineage (Tables S1 and S2). For ECC_ppuppp, a *pu* in *ty* and a *up* in *y* (sib/parent reverse; Figure S2C) could underlie the observation but is less probable than sib/parent reversals in *m* because the parent lineage is 50% shorter. In addition, in the assumed topology, no sib/p-sib parallel changes are consistent with this configuration. For ECC_ppuppp, the third most common configuration requires three *up* changes in the *ms*, *y*, and *eo* lineages, a sib/p-sib/gp-sib parallel scenario (gp-sib refers to a sibling of a grandparent; Figures S2D and S3D). The low occurrence of triple-hit codons (Table S2) results in less sensitivity to non-equilibrium scenarios for the *t* lineage than for the *m* lineage.

***ty* lineage:**  Inference on the *ty* lineage is complicated by a large number of ECC_SD possibilities. Parallel changes in both child lineages, *t* and *y*, can be interpreted as a single change in *ty*. Parallel changes in *ty* and *eo* are consistent with a single change in the ancestral *tyeo* lineage. Finally, parallel changes in *ty* and *ms* or *eo* and *ms* result in data consistent with a single change in *eo* or *ty*, respectively. Inference on *ty* is also complicated by a large number of reverse changes consistent with single-hit scenarios. Such changes can occur on the *ty*/*t*, *ty*/*y*, *tyeo*/*ty*, and *tyeo*/*eo* lineage pairs.

Extant codon configurations consistent with a single *pu* (ECC_ppuupp) and a single *up* (ECC_uuppuu) in the *ty* lineage are shown in Figures S4 and S5, respectively. Larger numbers of multiple-hit scenarios can underlie codon configurations consistent with single *up* and *pu* changes in the *ty* lineage (Figures S4C-E and S5C-E) than equivalent configurations for the *m* and *t* lineages. For the *ty* lineage, sib/parent reversals are shown in Figures S4E and S5E. Because the parental lineage, *tyeo*, is one third the length of *ms* and one half the length of *ty*, this scenario has a lower occurrence across simulations than sib/parent reversals underlying configurations consistent with single-hits for the *m* and *t* lineages. However, other multiple-hit scenarios are more common. Sib/p-sib parallel and child/child parallel scenarios are shown in Figures S4C, D and Figures S5C, D. For scenario C, one of the two changes occurs in the long *ms* lineage (sib/p-sib parallel). The probabilities of such scenarios are sensitive to departures from equilibrium; note the high probability of sib/p-sib parallel *up* changes under increased *N*_e_ (Table S2). In addition, inference on the *ty* lineage is complicated by substitutions on child lineages. If an unpreferred substitution occurs in the *ty* lineage, the probability for a reverse substitution in at least one of the two child lineages, *t* or *y,* can be relatively high when selection favors *up*. The ratio of ECC_ppuupp to ECC_uuppuu is lower than the ratios of equivalent configurations for the *m* and *t* lineages.

Figure S6 compares *d*_up,pu_ inference in the *m*, *t*, and *ty* lineages for MP and ML reconstructions. Inferred *d*_up,pu_ are similar in the three lineages under equilibrium (especially for ML inference; Figures S6A and D). The HKY85 parameterization allows quite accurate determination of the relative probabilities of single- and multiply-hit codons. However, differences in the magnitude of biases in *d*_up,pu_ inference are apparent in the non-equilibrium scenarios (Figures S6B, C, E, and F).

Parsimony biases are similar for the *m* and *t* lineages across the three scenarios (Figures S6A-C). The reduction of *d*_up,pu_ is slightly greater for the *m* lineage because *up* changes are underestimated to a greater degree (Table 1, Tables S1 and S2). Inference in the *ty* lineage appears to be somewhat less biased than in other lineages for the equilibrium and 1/3*N*_e_ cases but is more biased for 2*N*_e_. Inference on the *ty* lineage depends to a greater degree on contributions from multiply-hit codons than inference on the *m* and *t* lineages (Table 1, Tables S1 and S2). For the 2*N*_e_ scenario for MCU=0.9, the single change scenario underlies only half of ECC_ppuupp’s (*up* in *ms* + *up* in *eo* contributes 40% of these configurations). The apparent lack of bias in *d*_up,pu_ inference under MP for the *ty* lineage for the equilibrium and 1/3*N*_e_  cases reflects low inferred to actual values for *pu* changes that remain relatively flat with MCU. ML inference on the *ty* lineage is more strongly biased than inference on *m* and *t* for non-equilibrium scenarios. Overall, ML inference on the *t* lineage is the least sensitive to both biased codon usage and departures from equilibrium (inference bias differences are small for the 1x tree, however).


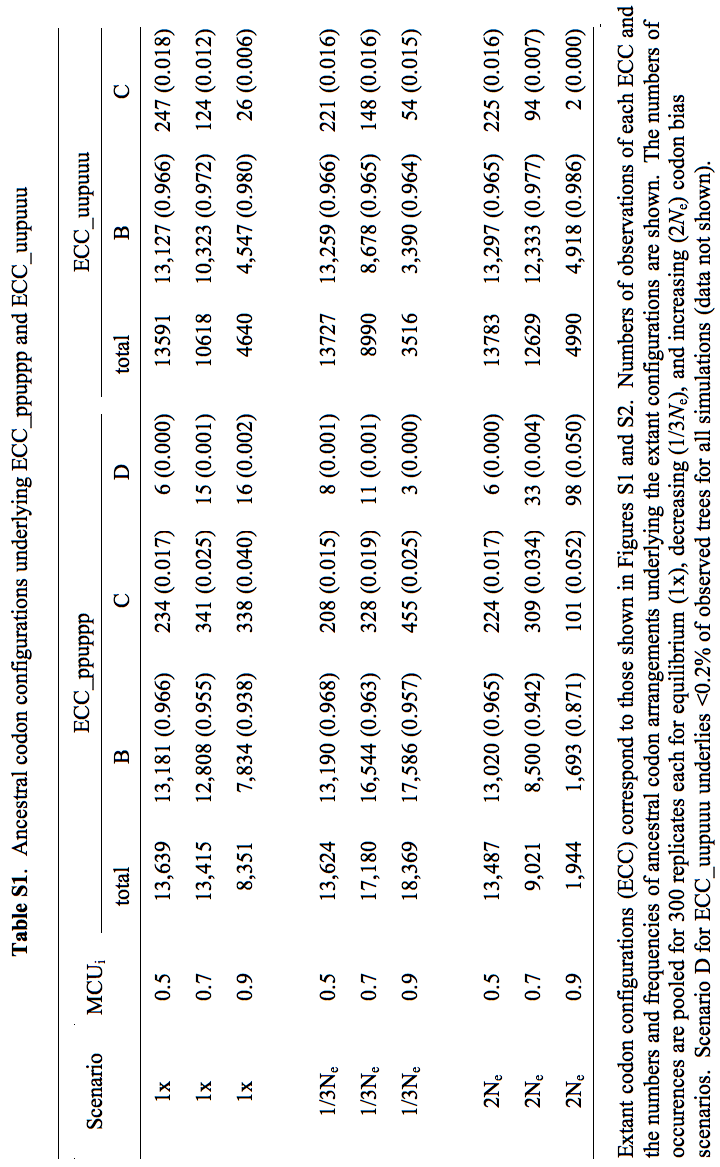

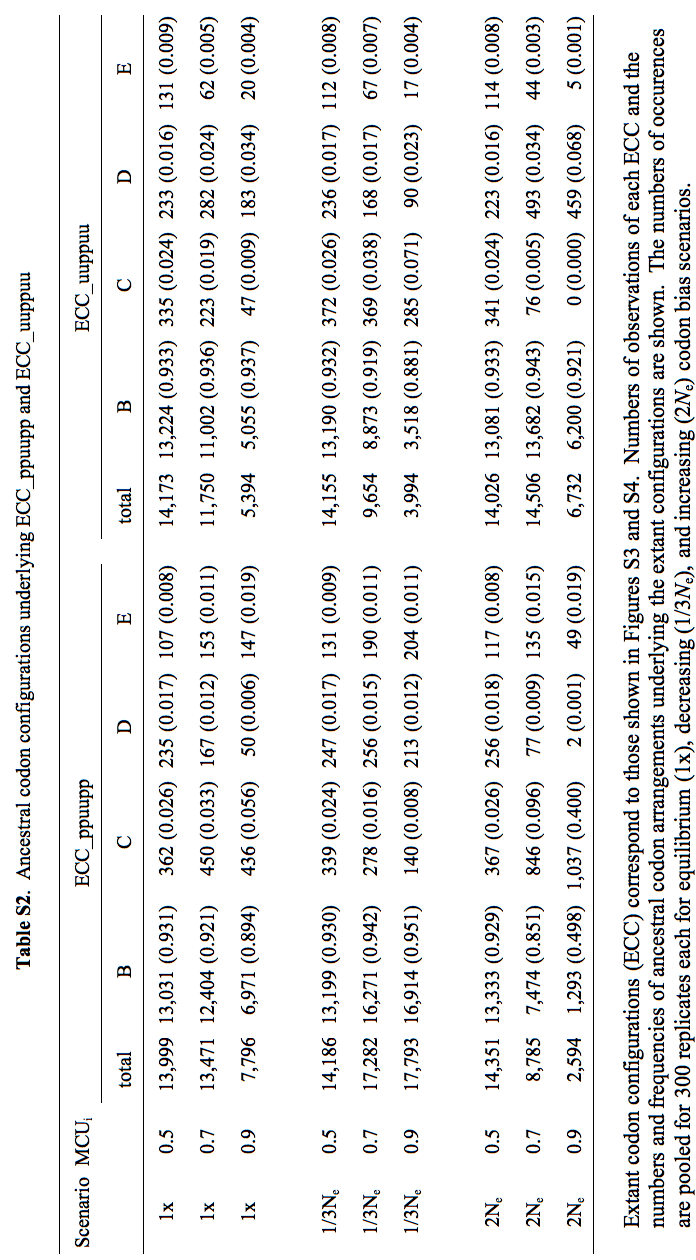


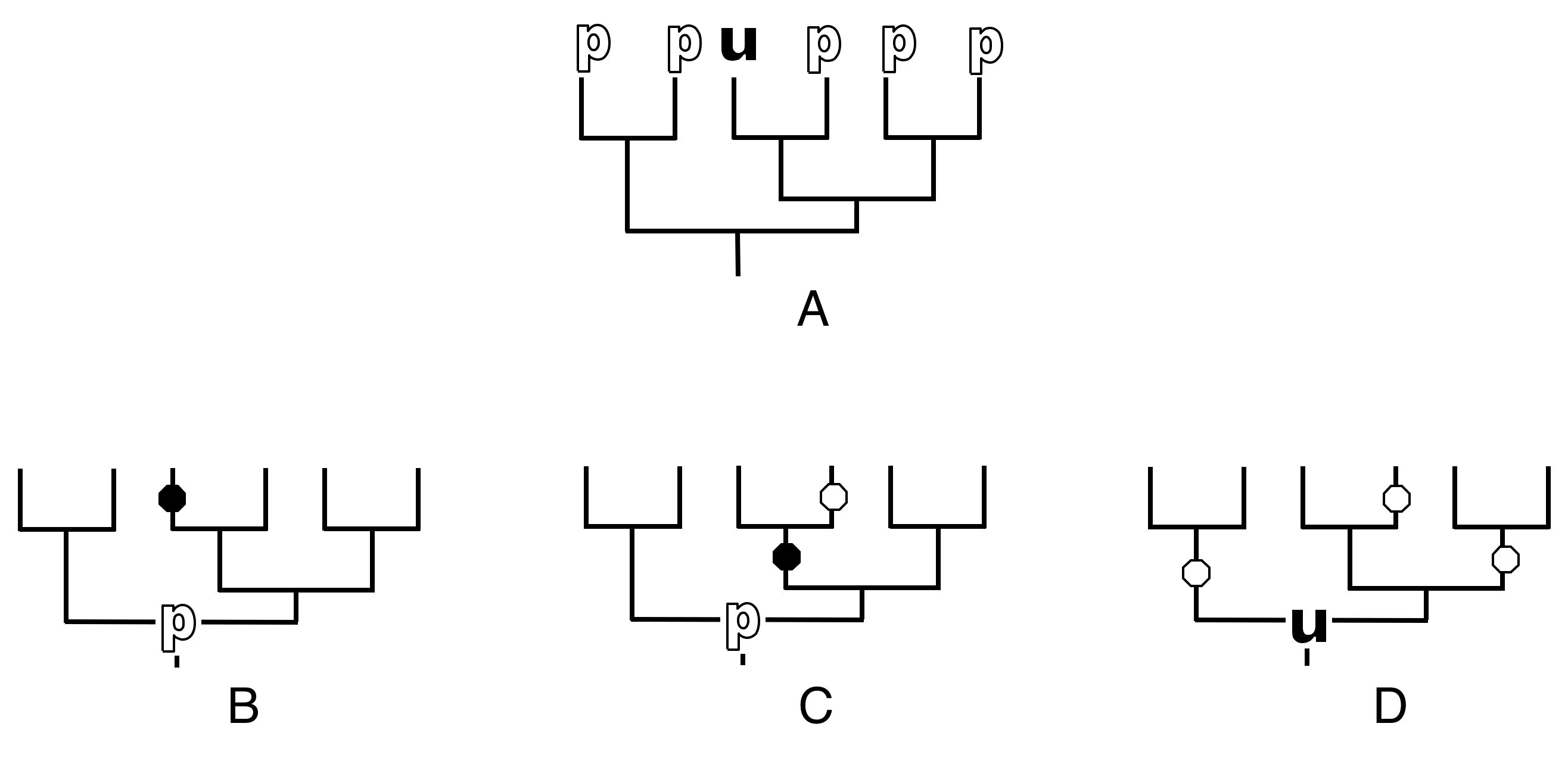


**Figure S2. Ancestral configurations for ECC_ppuppp.**  Common scenarios underlying the codon configuration shown in A are given in B, C, and D. “u” and “p” refer to unpreferred and preferred states, respectively. The ancestral state is shown at the *mstyeo* node for each scenario. C is a parent/child reverse scenario.


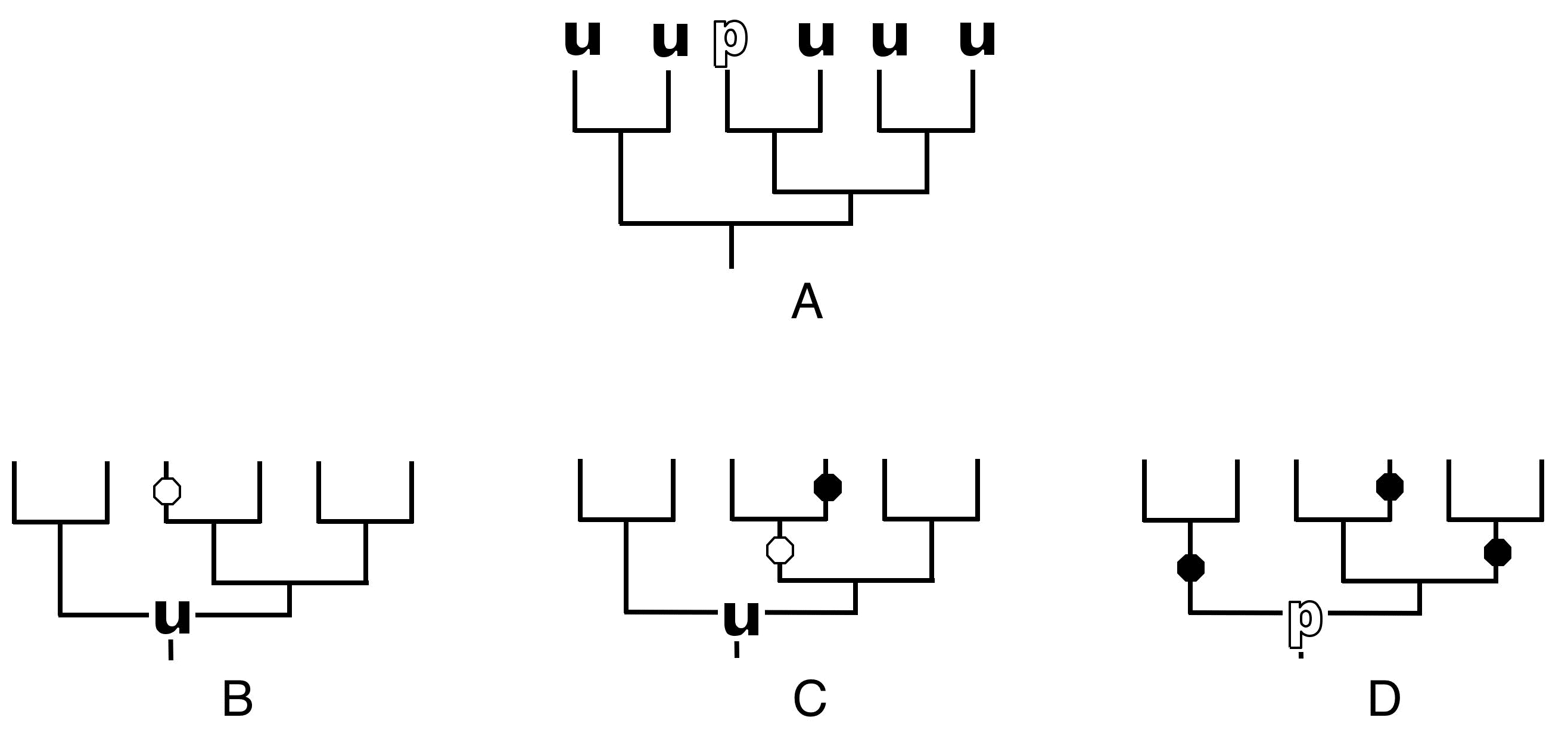


**Figure S3.**  **Ancestral configurations for ECC_uupuuu.**  Common scenarios underlying the extant codon configuration shown in A are given in B, C, and D. “u” and “p” refer to unpreferred and preferred states, respectively. The ancestral state is shown at the *mstyeo* node for each scenario.


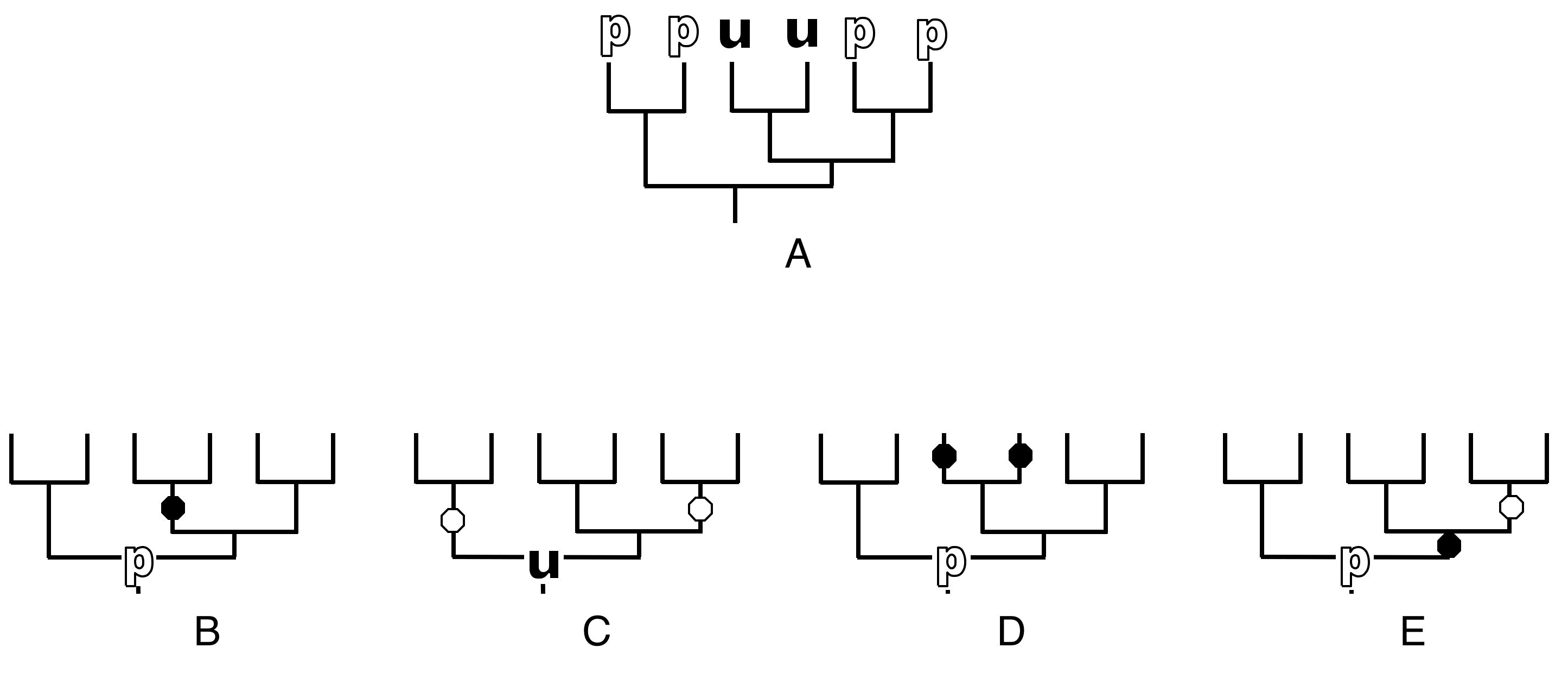


**Figure S4.**  **Ancestral configurations for ECC_ppuupp.**  Common scenarios underlying the codon configuration shown in A are given in B, C, D, and E. “u” and “p” refer to unpreferred and preferred states, respectively. The ancestral state is shown at the *mstyeo* node for each scenario. C is a sib/sib-ancestor parallel scenario, D is a child/child parallel scenario, and E is a sib/parent reversal scenario.


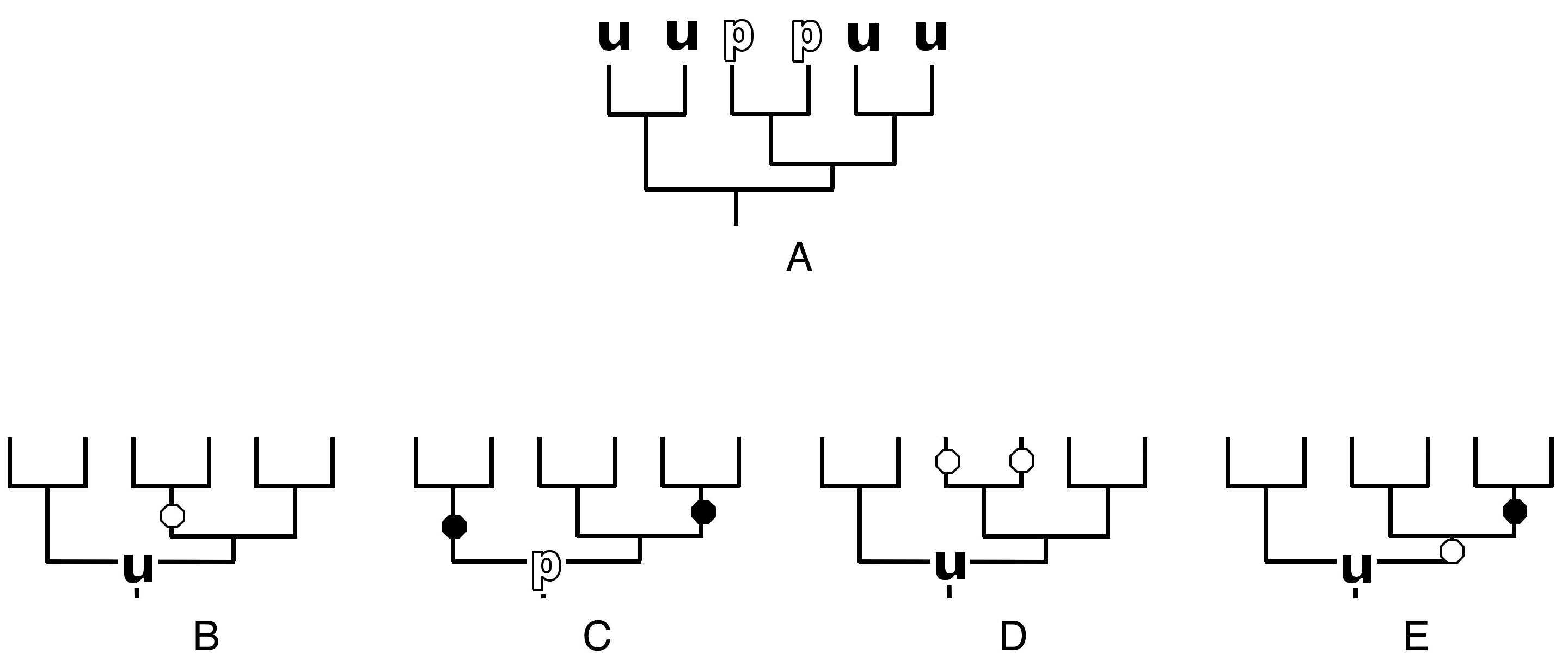


**Figure S5.**  **Ancestral configurations for ECC_uuppuu.** Common scenarios underlying the codon configuration shown in A are given in B, C, D, and E. “u” and “p” refer to unpreferred and preferred states, respectively. The ancestral state is shown at the *mstyeo* node for each scenario.


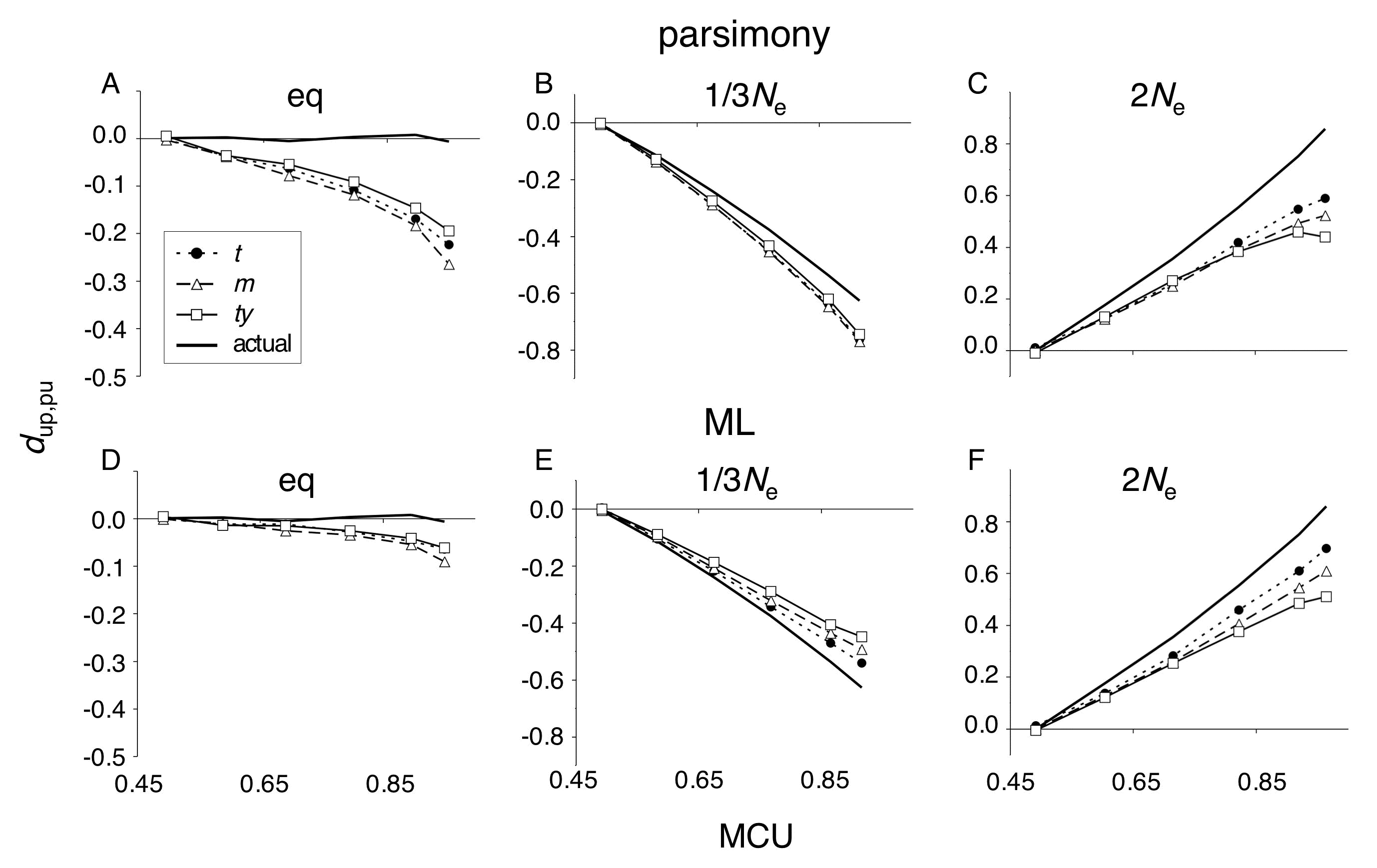


**Figure S6.** ***d*_up,pu_ inference in the *m*, *t*, and *ty* lineages under stationary and non-stationary codon bias evolution.**  *d*_up,pu_ values are averaged across 300 replicates. Graphs are shown for both parsimony and ML inference for the equilibrium, 1/3*N*_e_, and 2*N*_e_ simulations. The legend applies to all graphs. Curves for the *t* lineage are hidden below the *m* lineage data in B and below the *ty* lineage data in D. In the equilibrium scenario, ML is more reliable than MP for the *t* and *ty* lineages for MCU ≥ 0.6. Under decreasing codon bias (1/3*N*_e_), ML is more reliable than MP for the *t* lineage for MCU ≥ 0.6. However, MP is more reliable for the *ty* lineage for MCU ≥ 0.7. Under increasing codon bias (2*N*_e_), ML is more reliable than MP for the *t* lineage for MCU ≥ 0.7 and for the *ty* lineage for MCU ≥ 0.90. However, MP is more reliable for the *ty* lineage for MCU = 0.6.
