# Supplementary material for: Ancestral Inference and the Study of Codon Bias Evolution: Implications for Molecular Evolutionary Analyses of the Drosophila melanogaster Subgroup
Source: PLoS One. 2007 Oct 24;2(10):e1065. doi: 10.1371/journal.pone.0001065 (PMC2020436; doi:10.1371/journal.pone.0001065)
Supplement: Results S2 — Species composition and ancestral reconstruction biases. (0.64 MB DOC) [file pone.0001065.s004.doc]

Supporting Information for Akashi et al. “Ancestral inference and the study of codon bias….”

**Results S2** **Species composition and ancestral reconstruction biases**

Results so far have been shown for ancestral inference using simulated sequence data for six species given a topology and branch lengths mimicking those in the *D. melanogaster* subgroup. To determine ancestral reconstruction biases for studies using fewer sequences, inference biases on the *m* lineage were compared given sequences for all six species, three species (*m*, *s*, *y*) and four species (*m*, *s*, *y*, *e*). These configurations were chosen to emulate reconstructions that will be possible with available genome sequence data. Inferences for three and four species data were studied by deleting sets of sequences from the simulated data.

*d*_up,pu_ inference on the *m* and *s* lineages using the *msy* three-species set combines the *tyeo*, *ty* and *y* lineages as the sibling lineage to the parental *ms* lineage. Any parallel changes in either *m* or *s* and *tyeo*, *ty*, or *y* will result in ECC’s consistent with a single change in either *m* or *s*. Additional sequences from the *tyeo* clade would decrease the numbers of such configurations. For example, addition of data from the *t* lineage would result in parallel changes in *m* or *s* and *y* that are no longer consistent with a single change. Addition of data from either *e* or *o* would result in parallel changes in *m* or *s* and *ty* that are no longer consistent with a single change. The effect of species addition on inference biases are strongly dependent on departures from equilibrium on specific lineages in the tree. In particular, *d*_up,pu_ inference will be sensitive to parallel departures from equilibrium on the *m* or *s* and *tyeo*, *ty*, or *y* lineages.

Under MP, *d*_up,pu_ biases are quite similar for six, four, and three species data under equilibrium base composition and decreasing MCU (1/3*N*_e_) (Figure S7). Greater biases toward negative *d*_up,pu_ (underestimation of increases in MCU) were detected for four and three species comparisons for increasing MCU (2*N*_e_). For ML inference, additional sequences did not reduce *d*_up,pu_ bias for the equilibrium scenario, but biases were reduced for both the decreasing (1/3*N*_e_) and increasing (2*N*_e_) MCU scenarios. The enhanced accuracy of *d*_up,pu_ inference with larger species samples was large for high MCU genes in the 2*N*_e_ scenario. These results reflect the higher occurrence of parallel changes in the increasing than in the decreasing codon bias scenarios. For 1/3*N*_e_ simulations, among codons that experienced at least one *pu* change, 7.5% had multiple *pu* changes for MCU_i_=0.8. For 2*N*_e_ simulations, >25% of codons with a single *up* change had two or more parallel substitutions.


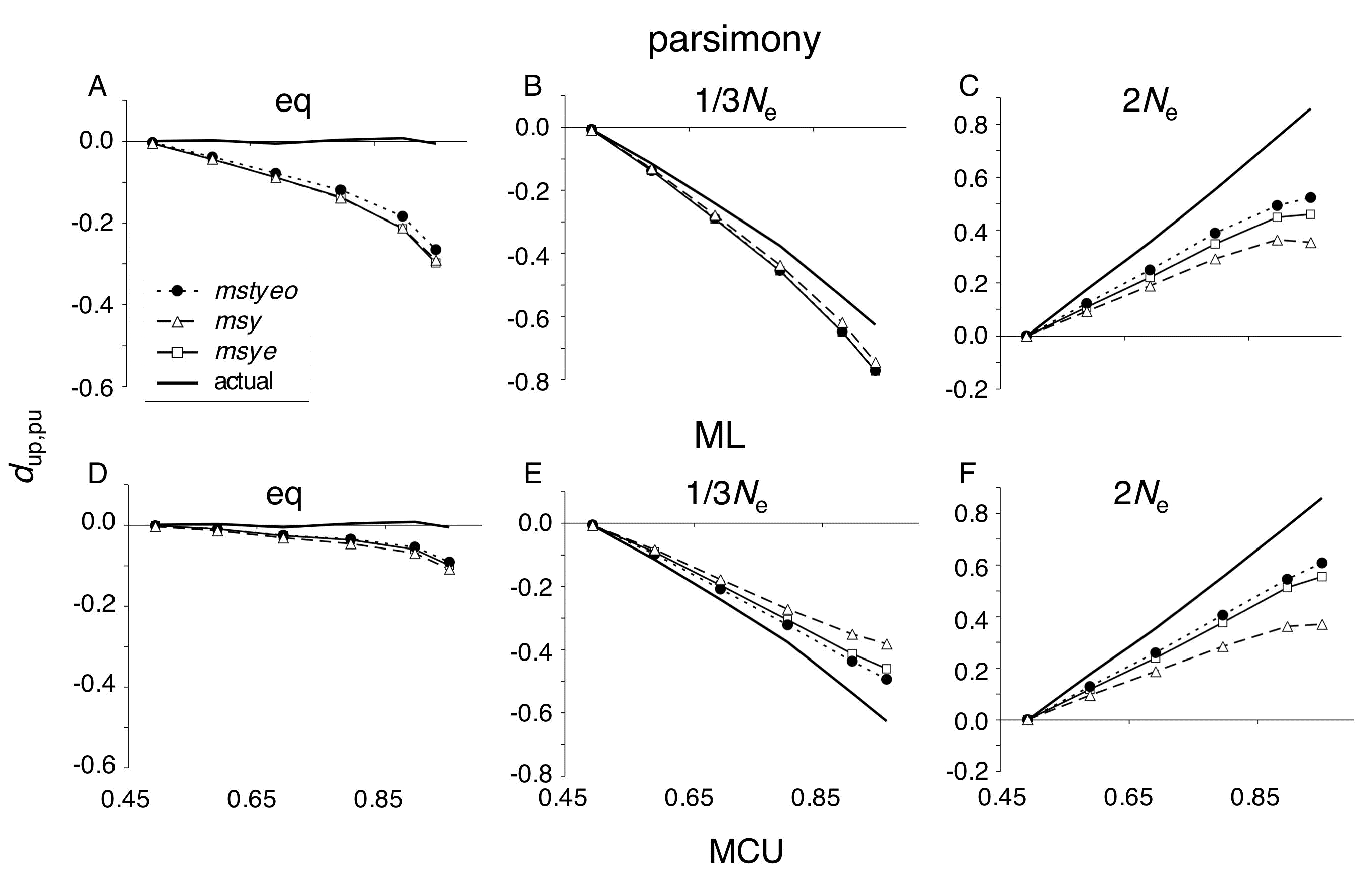


**Figure S7.** ***d*_up,pu_ inference on the *m* lineage using different species configurations.** *d*_up,pu_ values are averaged across 300 replicates. Graphs are shown for MP and ML inference for the equilibrium, 1/3*N*_e_, and 2*N*_e_ simulations. The legend applies to all graphs. X-axis values are identical for graphs in the same column.
